# Strategy for an Effective Eco-Optimized Design of Heteroleptic Cu(I) Coordination Polymers Exhibiting Thermally Activated Delayed Fluorescence

**DOI:** 10.1021/acs.inorgchem.3c01908

**Published:** 2023-11-27

**Authors:** Sabina W. Jaros, Jerzy Sokolnicki, Miłosz Siczek, Piotr Smoleński

**Affiliations:** Faculty of Chemistry, University of Wrocław, F. Joliot-Curie 14, 50-383 Wrocław, Poland

## Abstract

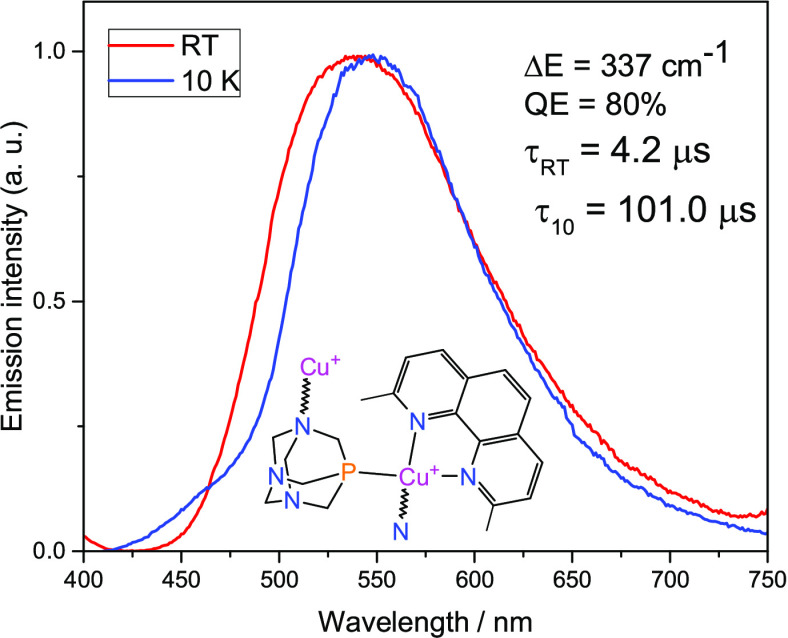

The new series of copper(I) coordination polymers [Cu(N–N)(μ-PTA)]_*n*_[PF_6_]_*n*_ {N–N = dmbpy (**1**), bpy (**2**), ncup (**3**), and phen (**4**)} were generated by straightforward reaction in solution or through a mechanochemical route, of [Cu(MeCN)_4_][PF_6_] with 1,3,5-triaza-7-phosphaadamantane (PTA) and the corresponding polypyridines, namely, 5,5′-dimethyl-2,2′-bipyridine (dmbpy), 2,2′-bipyridine (bpy), 2,9-dimethyl-1,10-phenanthroline (ncup), and 1,10-phenanthroline (phen). The compounds were obtained as air-stable solids and fully characterized by IR, NMR spectroscopy, and elemental analyses. The molecular structures were confirmed by single-crystal X-ray diffraction analysis (for **1**, **2**, and **4**), revealing infinite one-dimensional (1D) linear chains driven by μ-PTA *N,P*-linkers. All tested Cu(I) polymeric compounds show emission at room temperature, which was attributed to thermally activated delayed fluorescence (TADF). Evidence of the involvement of the excited singlet state in the emission process is presented. Comparing the photophysical properties of **1** and **2** as well as **3** and **4**, of which **1** and **3** have a stiffened structure, by introducing a methyl group to one of the ligands, we demonstrate how TADF properties depend on molecular rigidity. It is shown that stiffening of the structure reduces the flattening distortion around the Cu(I) center in the ^3^MLCT state. As a result, the Δ*E*(S_1_–T_1_) energy gap becomes smaller and the fluorescence quantum yield increases without significantly extending the emission lifetime. In particular, the Δ*E*(S_1_–T_1_) values for complexes **1** and **3** are among the shortest reported in the scientific literature, 253 and 337 cm^–1^, and the TADF lifetimes are τ(300 K) = 5.7 and 4.2 μs, respectively. The fluorescence quantum yields for these complexes are measured to be Φ_PL_(300 K) = 70 and 80%.

## Abstract 2

New copper(I) coordination polymers driven by aminophosphine and polypyridines were self-assembled via the LAG route, fully characterized, and showed intense luminescence activity, including TADF behavior.

## Introduction

Luminescent materials based on Cu(I)-coordination compounds have been receiving increasing attention due to their extensive field of biomedical imaging, chemical sensors, photocatalytic properties, biological labeling, and optoelectronic/display visualization devices.^1−5^ Especially, research in the field of thermally activated delayed fluorescence (TADF) demonstrated by organo-transition-metal complexes is of great interest due to the remarkable variability of emission properties.^6−9^

Among them, Cu(I) coordination compounds have recently been found to be promising inexpensive electroluminescent emitters because of their special photoluminescent properties, especially their potential to harvest both the singlet and triplet excitons for luminescence.^10,11^

The choice of the copper(I) cation in the design of efficient TADF emitters is crucial: The first organic light emitting diodes (OLEDs) used singlet excitons formed in fluorescent materials, giving an upper limit of 25% of the device’s efficiency due to spin statistics. “Second-generation” OLEDs use heavy metal complexes, such as iridium(III) compounds, in which the strong spin–orbit coupling (SOC) caused by the presence of heavy metal enables rapid intersystem crossing (ISC) and emission from the triplet state.^12−16^ It is a triplet harvesting mechanism. Such phosphorescent emitters allow 100% of the generated excitons to be collected to emit light but require heavy metals. As the resources of the rare metals used, such as iridium, are being finished, their prices are high. As a result, mass production of the OLEDs currently needs to be improved. An alternative strategy with high yields and the use of more abundant materials with low spin–orbital coupling, such as luminescent Cu(I) complexes, is thermally activated delayed fluorescence (TADF).^17−22^ These materials can also provide 100% of the device’s intrinsic quantum yield with triplet and singlet excitons in a process called the singlet harvesting mechanism. Hence, the luminescent material does not require a heavy metal inducing a strong SOC as the thermally activated singlet state usually carries sufficient allowedness with respect to the transition to the singlet ground state (spin allowed transition). Therefore, an efficient path for photon generation becomes available. The thermal S1 singlet state population from the lower triplet state T1 obviously requires a relatively small energy gap. Δ*E*(S_1_–T_1_) between these states. In practice, this means that efficient thermal activation with a fast reverse ISC is unlikely to occur for Δ*E*(S_1_–T_1_) significantly above 10^3^ cm^–1^ (≈130 meV). A small energy gap Δ*E*(S_1_–T_1_) in the coordination compounds of transition metals means the occurrence of a metal-to-ligand charge transfer (MLCT) state in which the frontier orbitals highest occupied molecular orbital (HOMO) and lowest unoccupied molecular orbital (LUMO) are spatially significantly separated from each other. Hence, the exchange interaction between the involved electrons is small, and the required minor splitting between the singlet S_1_ and triplet T_1_ state is achieved.^23−25^ However, a flattening distortion occurs for this kind of electronic excitation. This structural reorganization is the result of the formal oxidation of Cu(I) to Cu(II) upon excitation and is the reason for nonradiative luminescence quenching due to an increase of the Franck–Condon factors that couple the excited state and the ground state.^26−28^ Accordingly, the stiffening of the structure, which can be performed under sterically demanding ligands or by a rigid environment, leads to a higher quantum efficiency of photoluminescence and promotes a more excellent brightness.

The aromatic *N,N*-ligands such as 2,2′-bipyridine, 1,10-phenanthroline, and their various derivatives have been the subject of intense research due to their versatile coordination chemistry and recognized function in diverse biological systems.^29,30^ In this class of tetrahedral copper(I) compounds, N–N-ligands play an essential role in inducing the emission properties and concerning an application as organic light-emitting diodes (OLEDs) and light-emitting electrochemical cell (LEC) emitters, various phenanthroline, bipyridyl, and their derivative-based complexes have been published with diverse external quantum efficiencies in electroluminescent devices.^31−42^

On the one hand, copper(I) complexes are usually synthesized under mild conditions as opposed to expensive Ir(III) compounds, obtained at relatively high temperatures and giving mixtures of homo- and heteroleptic species, which even occurs when processing the compounds after isolation.^43,44^

On the other hand, many copper(I) compounds are not air-stable and are vulnerable to oxygen and water, compared to Cu(II) complexes; therefore, ligands or coligands stabilizing the low oxidation state of metal center play a vital role in the application in OLED/LEC devices.^45^

In this regard, the air-stable and water-soluble aminophosphine 1,3,5-triaza-7-phosphaadamantane (PTA) represents an excellent alternative to conventional phosphorus(III) ligands, stabilizing low oxidation state of transition metal in coordination compounds.^46−50^

For this reason, the application of the hybrid—1,3,5-triaaza-7-phosphaadamantane (PTA) as a water-stable cagelike *P,N*-building block as well as photo functional-aromatic *N,N*-ligands offers a perspective way toward the preparation of novel Cu(I)-coordination networks as air- and water-stable TADF emitter.

Thus, we now report the liquid-assisted grinding (LAG) route generation and full characterization of the series of new coordination polymers [Cu(N–N)(μ-PTA)]_*n*_[PF_6_]_*n*_ {N–N = dmbpy (**1**), bpy (**2**), ncup (**3**), and phen (**4**)} which constitute the first examples of Cu(I)-PTA derivatives containing polypyridine ligands. Additionally, **1**–**4** were also evaluated for their advanced photoluminescent properties. Especially in the compounds we studied, the problem of molecular flattening after electronic excitation is addressed by ligands with high steric requirements. For example, sterically demanding groups at positions 2 and 9 of 1,10-phenanthroline may effectively inhibit the tendency to flatten in complexes Cu(I).^51,52^ By using this ligand (methyl groups) in polymeric compounds, which themselves show a specific stiffness, it is possible to stiffen the molecular structure of the complex additionally. The same compounds were examined for comparison but without the sterically demanding groups to confirm the assumed effect. It will be shown that the title compound solves the problem discussed above.

## Results and Discussion

### Synthesis

**Figure 1:**
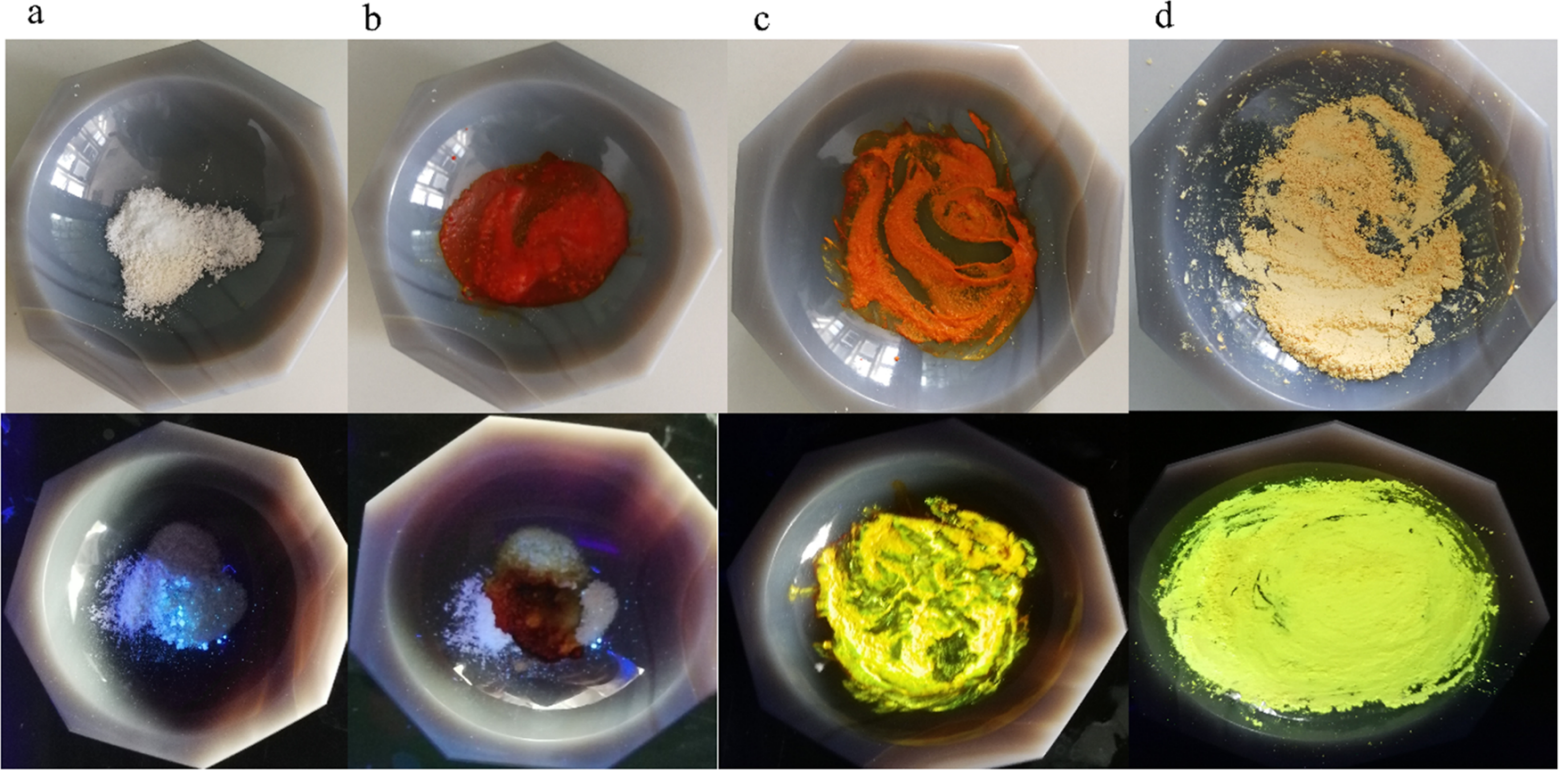
UV monitoring of the LAG synthesis of **3** (down views; the top images obtained under daylight): (a) a dry mixture of [Cu(MeCN)_4_][PF_6_] with ncup and PTA in 1:1:1 molar ratio; (b) the mixture with a few drops of MeCN; (c) mixture after 30 s of grinding; (d) ground reaction mixture after 1 min.

**Scheme 1:**
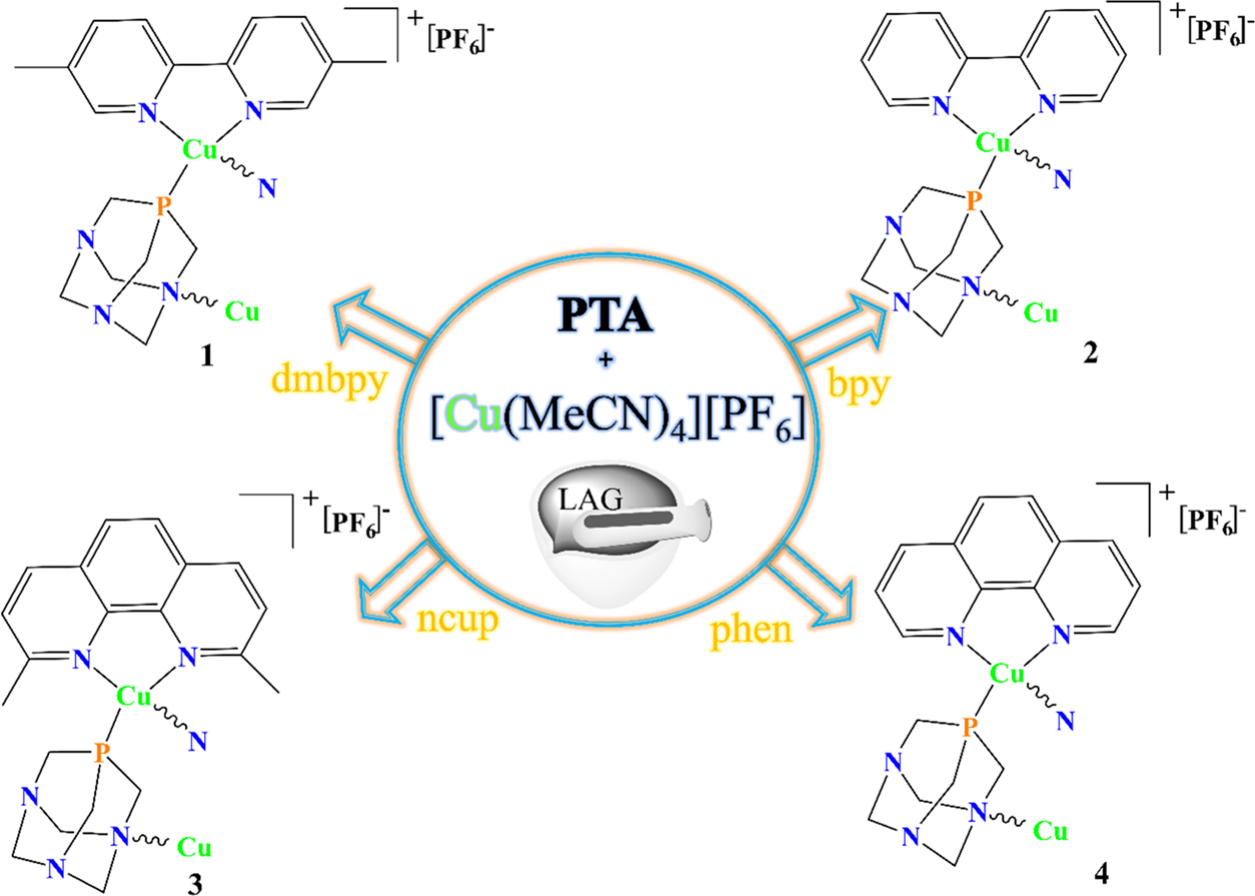
Schematic Representation of the Mechanochemical Synthesis of Compounds **1**–**4** and Ligand Connectivity

Green synthetic methods for preparing a functional coordination polymer and metal–organic framework (MOF) are particularly interesting due to environmental and economic aspects. Thus, through a selection of heteroleptic ligands, *N*,*N*-polypyridines and 1,3,5-triaza-7-phospaadamantane were synthesized herein, mechanochemically, using LAG, a series of luminescent active copper(I) coordination polymers. All four Cu(I) compounds with a general formula [Cu(N–N)(μ-PTA)]_*n*_[PF_6_]_*n*_ were successfully synthesized with high yields. Their synthesis involves simply utilizing a pestle and mortar and applying an η range of 0.82–0.90 μL·mg^–1^. A stoichiometric 1 min LAG reaction of [Cu(MeCN)_4_][PF_6_], PTA, and sterically hindered N–N-ligand (ratio 1:1:1) yields a series of new Cu(I) coordination polymer driven by *P,N*-coordinated linker (Scheme 1, Figures 1 and S24–S26). The self-assembly LAG syntheses of **1**–**4** are highly reproducible and can be easily scaled up. Each reaction was repeated three times via the above-mentioned method. The desired compounds **1**–**4** were obtained each time with high yields (yield >84%) without undesirable byproducts, confirming their excellent reproducibility. The purity of the compounds was confirmed by elemental and spectroscopic analyses (see the Experimental Section) as well as powder X-ray diffraction (PXRD) analysis for compounds **1**, **2**, and **4** (Figures S4–S6).

Moreover, as described herein, the LAG synthetic route is fast, simple, and energy-efficient, minimally affecting its environmental impact. Alternatively, compounds **1**–**4** can be synthesized using traditional wet synthesis, with a much lower yield of up to 40%. Moreover, during the crystallization process, after the classic wet syntheses, the formation of a byproduct with the general formula [Cu(PTA)_4_][PF_6_]·MeCN was observed, which significantly decreased the final yield and purity of the main products.

The products **1**–**4** have been isolated as air- and moisture-stable, yellow (**1**–**3**) or orange (**4**) microcrystalline solids and characterized by FT-IR, ^1^H, and ^31^P{^1^H} NMR spectroscopy, elemental analyses, and single-crystal/powder X-ray diffraction (for **1**, **2**, and **4**). A noteworthy feature of **1**–**4** concerns their hydrosolubility, with the *S*_25°C_ values ranging from 1 to 5 mg·mL^–1^. In addition, these compounds are soluble and relatively stable in dimethyl sulfoxide (DMSO), MeCN, and MeOH (see the Experimental Section).

The FT-IR spectra of **1**–**4** revealed characteristic stretches of coordinated PTA linker and N–N-polypyridine ligands and confirmed the general formula of [Cu(N–N)(μ-PTA)]_*n*_[PF_6_]_*n*_ compounds. Strong and medium ν(C–C, C–H, C–N, C–P) bands are detected in the 1600–500 cm^–1^ region and confirm the coordination of the PTA spacer and N–N-ligand to the metal center (Figures S7–S10).^53^ Other strong vibrations appeared in the spectra in the 839–837 cm^–1^ range, indicating the presence of the PF_6_^–^ counterion.

The ^1^H NMR spectra of **1**–**4** in DMSO-*d*_6_ solutions exhibit a set of typical resonances due to the methylene protons of coordinated PTA and polypyridines, slightly shifted in comparison with the corresponding uncoordinated ligands (Figures S11–S14).^29−31,53^ The ^31^P{^1^H} NMR spectra of **1**–**4** show broad singlets in the δ range from −73.3 to −94.1 ppm, which are shifted downfield in comparison with the uncoordinated PTA (Figures S15–S18).^53^ Also, the spectra reveal characteristic septets at δ −144.0 ppm due to [PF_6_]^−^ anions. The NMR data generally support preserving the coordination environments around Cu^I^ atoms upon the dissolution of **1**–**4** in the deuterated solvents.

### Crystal Structures

**Figure 2:**
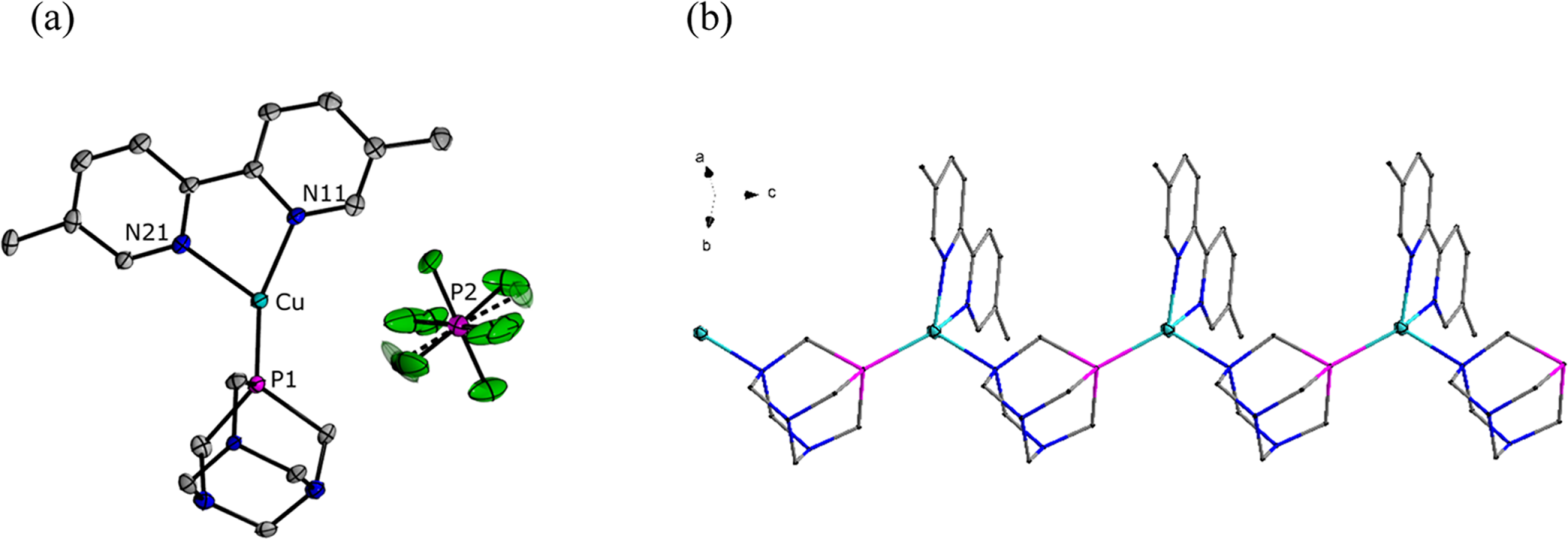
(a) Asymmetric part of the structure of compound **1**. (b) Part of the crystal structure of compound **1** showing 1D coordination polymer chain driven by μ-PTA spacer, where N, P, and Cu are blue, purple, and aquamarine, respectively. H atoms and the [PF_6_]^−^ anion are omitted for clarity.

Single-crystal X-ray studies confirmed that **1**, **2**, and **4** exist in the solid state as one-dimensional (1D) coordination polymers. The crystal structure of **1** was assigned to the noncentrosymmetric *Pna2*_1_ space group in the orthorhombic crystal system. The asymmetric part of the unit is built of one [Cu(dmbpy)(PTA)]^+^ cation and one [PF_6_]^−^ anion (Figure 2a). The copper(I) ion is four-coordinated, whereas the coordination sphere is filled with three nitrogen atoms (two from the dmbpy ligand and one from the PTA ligand) and one phosphorus atom of another PTA cage. As a result, the PTA ligand acts as a linker between copper atoms. The copper ions and PTA ligands are alternately arranged to form a 1D structure along *c* axis (Figure 2b). [PF_6_]^−^ anions are disordered over two positions and are located in channels formed between copper coordination polymer chains.

**Figure 3:**
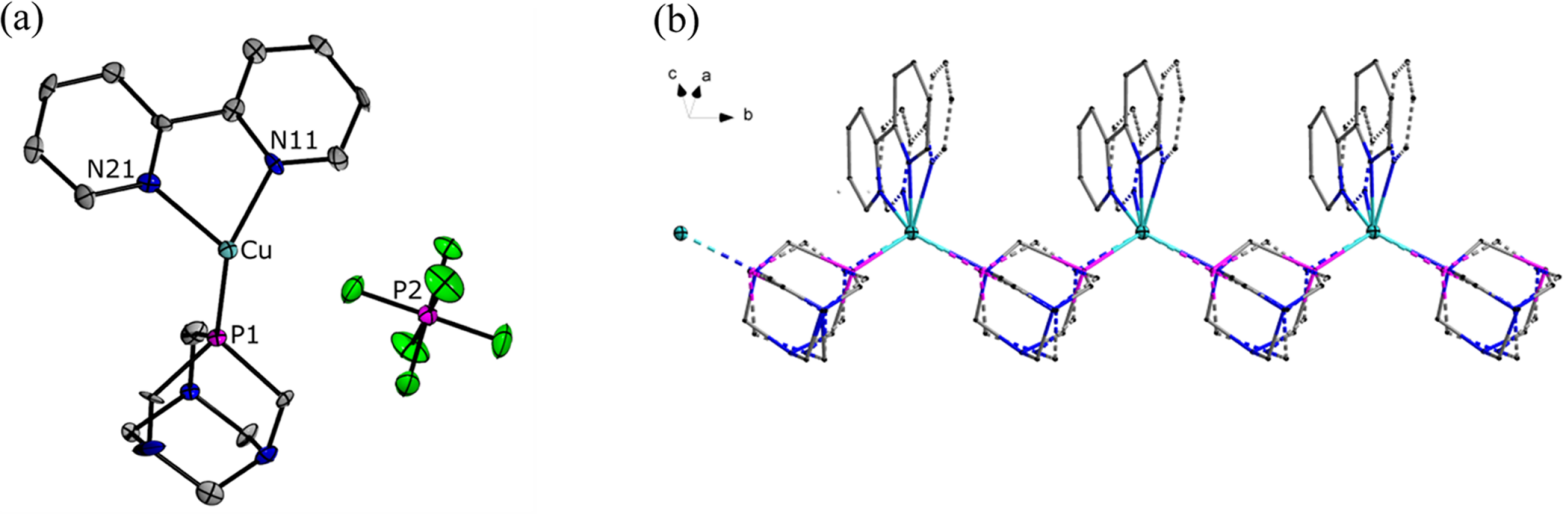
(a) Asymmetric part of the structure of compound **2**. (b) Part of the crystal structure of compound **2** showing 1D coordination polymer chain driven by μ-PTA spacer, where N, P, and Cu are blue, purple, and aquamarine, respectively. The disordered components are drawn using full and broken lines. H atoms and [PF_6_]^−^ anion are omitted for clarity.

**Figure 4:**
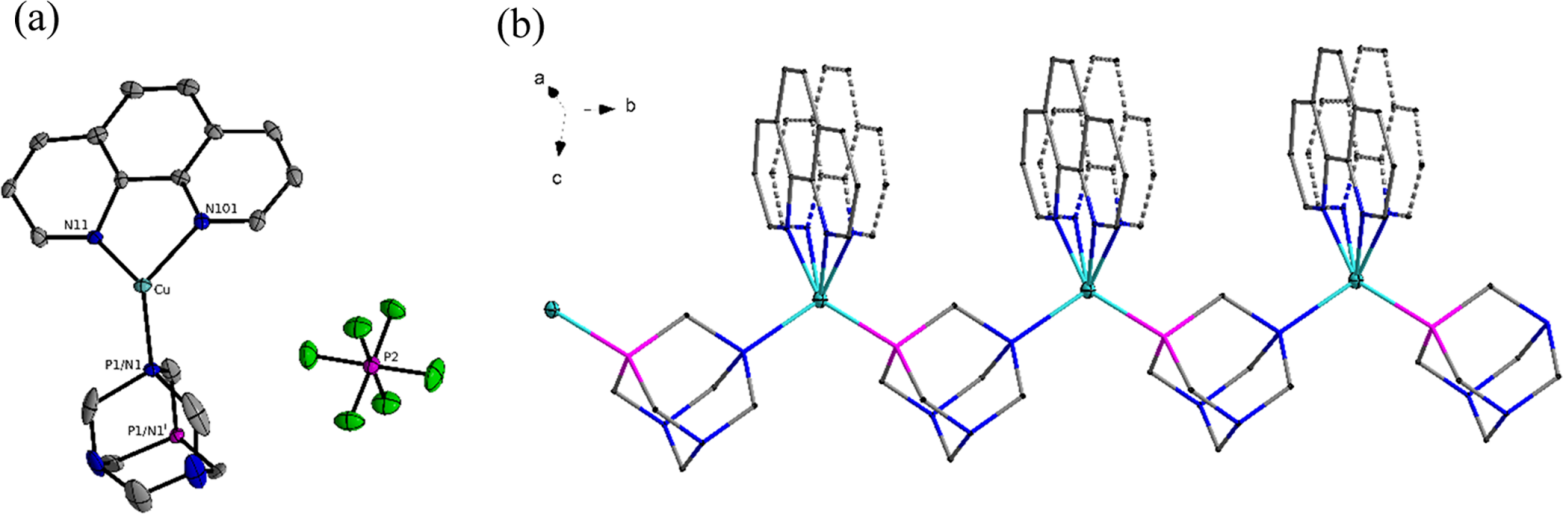
(a) Coordination environment of compound **4**. (b) Part of the crystal structure of compound **4** showing 1D coordination polymer chain driven by μ-PTA spacer, where N, P, and Cu are blue, purple, and aquamarine, respectively. The disordered components are drawn by using full and broken lines. H atoms and [PF_6_]^−^ anion are omitted for clarity. Symmetry code: (i) *x*, 3/2 – *y*, *z*. The PTA is disordered over two positions with an occupation factor of 0.5.

The structures of **2** and **4** crystallize in an orthorhombic *Pnma* space group and are isomorphous, with very similar unit cells; therefore, only structure **2** will be described. The asymmetric part of crystal **2** is composed of one [Cu(bpy)(PTA)]^+^ cation and one [PF_6_]^−^ anion {Figure 3a (**2**), 4a (**4**)}. The coordination geometry of the copper site can be described as disordered tetrahedral with two nitrogen atoms from the bpy(**2**)/phen(**4**) ligand, one nitrogen, and one phosphorus atom from the PTA ligand. The PTA ligand plays a key role by linking two copper atoms in creating a 1D polymer chain. From the other side, bpy(**2**)/phen(**4**) ligands coordinated to copper atoms create a ladder-like structure perpendicular to the *b* axis (Figures 3b and 4b).

### Photophysical Properties of **1**–**4**

**Figure 5:**
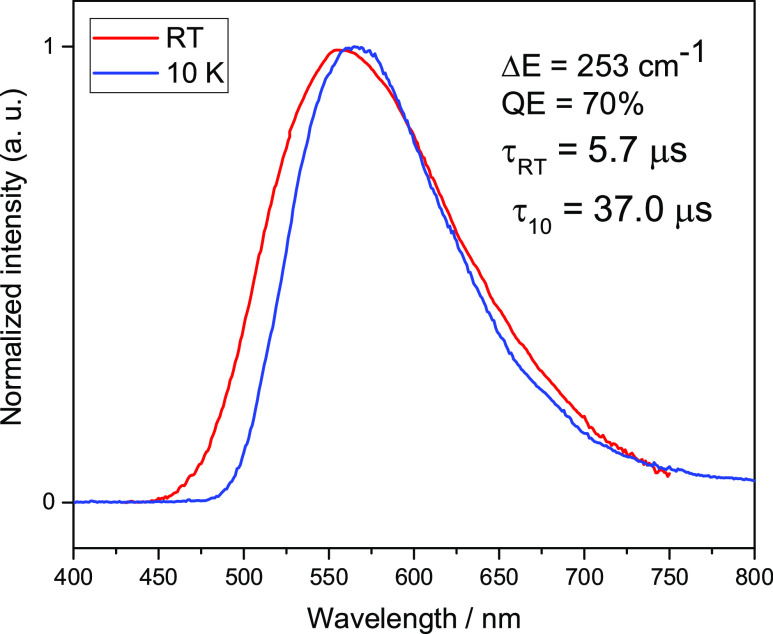
Normalized luminescence spectra of **1** powder recorded at ambient (red traces) and 10 K (blue traces) temperatures. λ_exc_ = 375 nm.

**Figure 6:**
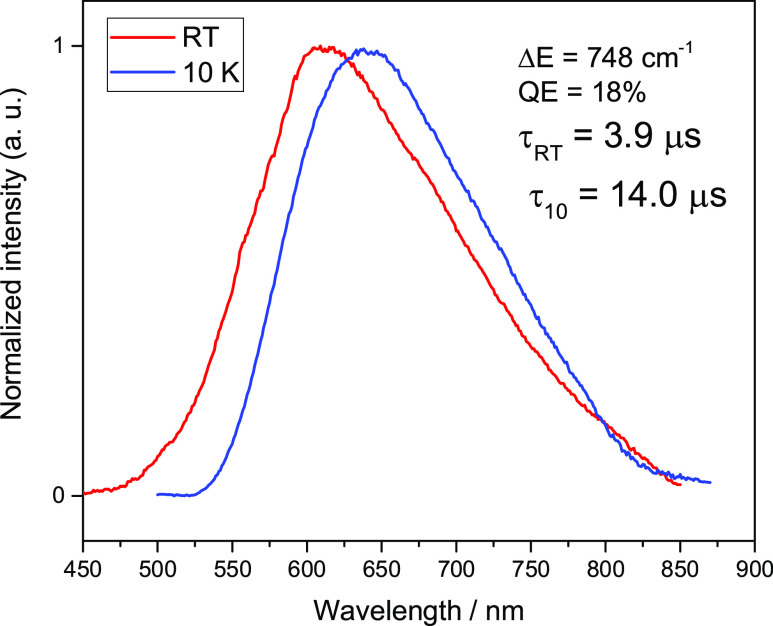
Normalized luminescence spectra of **2** powder recorded at ambient (red traces) and 10 K (blue traces) temperatures. λ_exc_ = 375 nm.

Photophysical data of the four compounds at ambient temperature and 10 K are summarized in Table 1. All tested complexes show intense luminescence when excited with UV light. The corresponding transitions are assigned to be of ^1,3^MLCT character. Figures 5 and 6 show the emission spectra for complexes **1** and **2** at room temperature and 10 K. Unnormalized luminescence spectra are provided in the Supporting Information as Figures S19–S22. The temperature of 10 K is assumed to be low enough to attribute an emission to phosphorescence. Compound **1** at 10 K shows emission in the spectrum’s green-yellow range with a maximum of 566 nm and a decay time of τ(10 K) = 67 μs. At 300 K, the maximum emission shifts to the shorter wavelengths by 9 nm, and the decay time is reduced to τ(300 K) = 5.7 μs. At the same time, the radiative rate constant increases 12 times from 1.0 × 10^4^ to 1.2 × 10^5^ s^–1^. Δ*E*(S_1_–T_1_), in this case, is extremely small and amounts to only 253 cm^–1^, one of the smallest values reported so far. This means that the decay time τ(S_1_) cannot be shorter than a few microseconds due to the proximity of the opposite parity state (T_1_), but it is still acceptably short. It should be noted that all luminescence decay curves at 300 K are biexponential, so the values presented are a weighted average (see SI Figure S23 and short explanation for more details).

**Table 1:** Luminescence Properties of **1**–**4**

compound/parameter	**1**	**2**	**3**	**4**
Δ*E*(S_1_–T_1_) (cm^–1^)	253	748	337	1300
λ_max_(300/10 K) (nm)	557/566	612/640	540/553	592/640
Φ_PL_(300 K) (%)	70	18	80	20
τ(300 K) (μs)	5.7	3.9	4.2	1.1
τ(T_1_ → S_0_) (μs)	67.0	14.0	101.0	39.7
τ(S_1_ → S_0_) (ns)	233	50	274	0.77
*k*^r^(S_1_ → S_0_) (s^–1^)	1.2 × 10^5^	4.6 × 10^4^	1.9 × 10^5^	1.8 × 10^5^
*k*^r^(T_1_ → S_0_) (s^–1^)	1.0 × 10^4^	1.3 × 10^4^	7.9 × 10^3^	5.0 × 10^4^

This behavior shows that at room temperature, the emission from a higher state is activated and that this transition is much more allowed than low-temperature phosphorescence. In combination with the observed blue shift of the spectrum after increasing the temperature to 300 K indicates the presence of TADF. For compound **2**, the emission at 10 K shows a maximum at 640 nm which shifts to 612 nm at 300 K. This gives Δ*E*(S_1_–T_1_) = 748 cm^–1^, thus the average gap size, and the quantum efficiency of S_1_ emission, hence TADF, is smaller and amount to 18%. The decay time of S_1_ emission at this Δ*E*(S_1_–T_1_) is τ(300 K) = 3.9 μs, clearly shorter than that at a low temperature, τ(10 K) = 14 μs.

The differences in the photochemical properties of both compounds result from the presence of methyl groups in the pyridine rings of compound **1**. The electron-donating −CH_3_ substituents on the pyridine rings not only stiffen the structure through a steric effect but also promote changes in polarity that affect the crystal packing.^54^ This results in less geometrical distortion, so the energy level of HOMO does not increase too much. Unlike compound **1**, the HOMO energy of compound **2** increases substantially due to the considerable geometrical distortion. This obviously impacts a significant reduction of the QY of the emission of compound **2** and shifts its emission to longer wavelengths.

**Figure 7:**
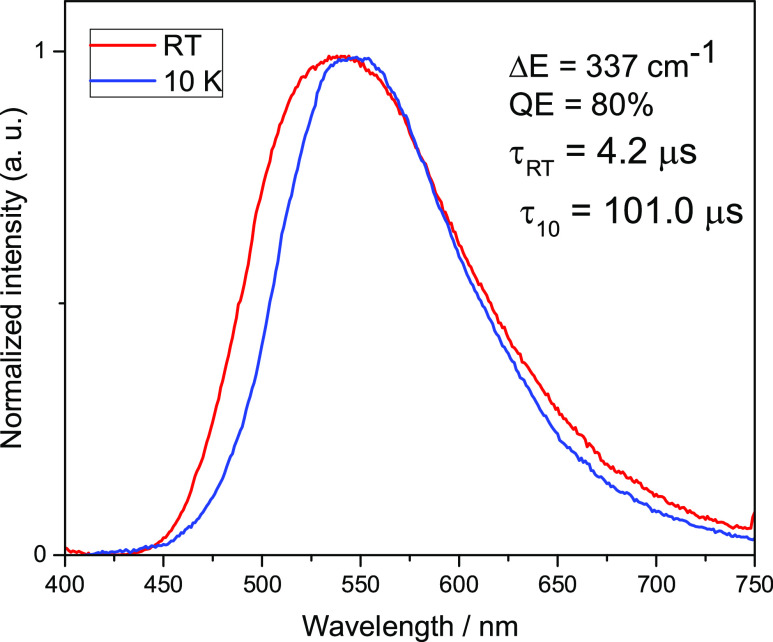
Normalized luminescence spectra of **3** powder recorded at ambient (red traces) and 10 K (blue traces) temperatures. λ_exc_ = 375 nm.

**Figure 8:**
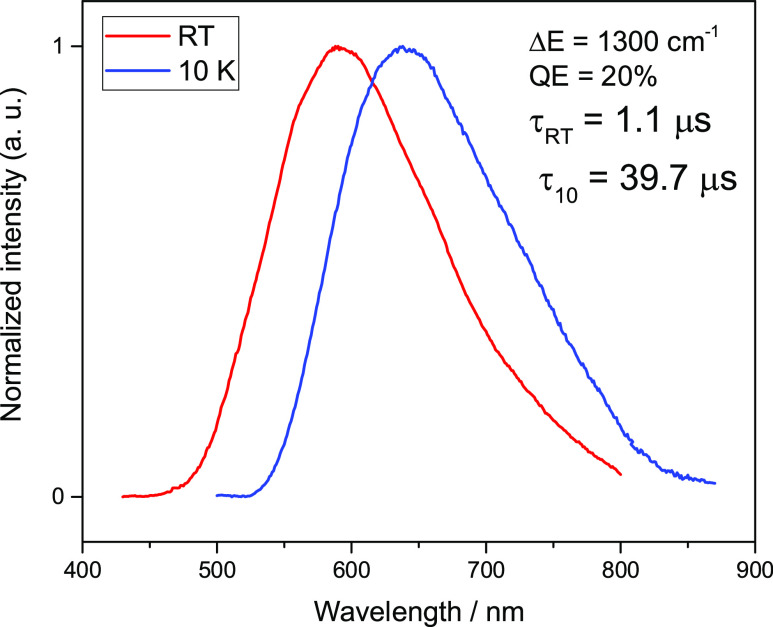
Normalized luminescence spectra of **4** powder recorded at ambient (red traces) and 10 K (blue traces) temperatures. λ_exc_ = 350 nm.

Similar conclusions can be drawn from a comparison of the photophysical properties of compounds **3** and **4**. The respective emission spectra for complexes **3** and **4** at room temperature and 10 K are shown in Figures 7 and 8. The emission at 10 K of compound **3**, which contains methyl groups in phenanthroline rings, is characterized by a band with a maximum at 553 nm and decay time τ(10 K) = 101.0 μs. Increasing the measurement temperature to 300 K shifts the emission maximum to 540 nm and shortens the emission decay time to τ(300 K) = 4.2 μs. Δ*E*(S_1_–T_1_) is, in this case, only 337 cm^–1^ and the QY (300 K) of the emission is 80%. The absence of methyl groups on the pyridine rings (compound **4**) results, as for compound **2**, in a shift of the emission toward higher energies, 640 nm at 10 K and 592 nm at 300 K. Significant flattering distortion causes the energy gap Δ*E*(S_1_–T_1_) to be very large and amounts to 1300 cm^–1^. At the same time, the decay time is reduced from τ(10 K) = 39.7 μs to τ(300 K) = 1.1 μs. This short decay time at ambient temperature results from such a large energy gap and the lack of interaction between the S_1_ and T_1_ states with opposite parity. The emission quantum yield of compound **4** (20%) is four times lower than that of compound **3** (80%).

It is worth noting that the small spatial overlap of HOMO and LUMO also results in a smaller strength of the S_1_ → S_0_ transition oscillator. Thus, it should be expected that as Δ*E*(S_1_–T_1_) decreases, singlet decay time τ(S_1_) increases. Indeed, we can see this trend by comparing the compounds **1** (τ(S_1_ → S_0_) = 233 ns, Δ*E*(S_1_–T_1_) = 253 cm^–1^) and **2** (τ(S_1_ → S_0_) = 50 ns, Δ*E*(S_1_–T_1_) = 748 cm^–1^) as well as **3** (τ(S_1_ → S_0_) = 274 ns, Δ*E*(S_1_–T_1_) = 337 cm^–1^ and **4** (τ(S_1_ → S_0_) = 0.77 ns, Δ*E*(S_1_–T_1_) = 1300 cm^–1^). Similar observations were made previously.^55^

A consequence of the long internal singlet decay time is the absence of prompt fluorescence. Compared to singlet decay times of a few 100 ns, the S_1_ → T_1_ transition is much faster, on the order of 10 ps, and thus represents the dominant decay path. Consequently, at low temperatures, the S_1_ state is mostly depopulated by ISC before prompt fluorescence can occur.^56,57^

## Conclusions

In summary, we described herein a fast, simple, and energy-efficient self-assembly LAG synthetic method for a new copper(I)-based coordination polymers type [Cu(N–N)(μ-PTA)]_*n*_[PF_6_]_*n*_ {N–N = dmbpy (**1**), bpy (**2**), ncup (**3**), and phen (**4**)}. The products have been isolated as air- and moisture-stable, microcrystalline solids and characterized by FT-IR, ^1^H, and ^31^P{^1^H} NMR spectroscopy, ESI^+^-MS, elemental analyses, and single-crystal/powder X-ray diffraction. A noteworthy feature of **1**–**4** concerns their hydrosolubility and stability in aqua-media, In addition, these compounds are soluble in DMSO, MeCN, and MeOH. All tested compounds show luminescence at room temperature. It has been shown to be thermally activated delayed fluorescence (TADF). Cu(I)-based TADF emitters usually suffer from nonradiative deactivations since strong geometry changes (flattering distortion) occur upon excitation. In this research, we have shown a way to stiffen the structure of the Cu(I) coordination polymers at the molecular level, significantly reducing the fluttering distortion around the Cu(I) center. Comparative studies of two pairs of Cu(I) polymeric compounds, where one ligand in each pair was additionally stiffened with methyl groups, showed that such stiffening of the structure leads to a significant reduction of the Δ*E*(S_1_–T_1_) energy gap and a 4-fold increase in fluorescence quantum efficiency. Specifically, the internal emission quantum yield for compounds **1** and **3** containing ligands 5,5′-dimethyl-2,2′-bipyridine (dmbpy) and 2,9-dimethyl-1,10-phenanthroline (ncup) is Φ_PL_(300 K) = 70 and 80%, respectively. Together with the relatively short emission decay times of the thermally activated delayed fluorescence of only a few microseconds, these complexes may qualify as desirable candidates for singlet-harvesting emitters in OLEDs.

Finally, it should be noted that these systems are also of interest for other applications in various types of molecular electronic devices (e.g., LEC—light-emitting electrochemical cell) because of their photochemical behavior. Therefore, the extension of the work toward the application of described Cu(I) compounds as potent LEC devices is currently in progress and will be reported elsewhere.

## Experimental Section

### Chemicals

All standard chemicals and solvents were obtained from commercial suppliers. PTA (1,3,5-triaza-7-phosphaadamantane)^58,59^ and [Cu(MeCN)_4_][PF_6_]^60^ were synthesized according to published methods.

### Analytical Methods

Bruker IFS 1113v (Germany) or BIO-RAD FTS3000MX (BIO-RAD, France) instruments (range 4000–400 cm^–1^) were used to measure the IR spectra (abbreviations: vs, very strong; s, strong; m, medium; w, weak; br., broad). NMR spectra were recorded in DMSO-*d*_6_ solvent using a Bruker 500 AMX spectrometer (Bruker BioSpin MRI GmbH, Germany) at ambient temperature (abbreviations: s, singlet; d, doublet; t, triplet, br., broad). ^1^H chemical shifts (δ) are expressed in ppm relative to Si(Me)_4_, while δ(^31^P) shifts are relative to an external H_3_PO_4_ (85% aqueous solution). The Elemental Analyzer Vario ELCube (Elementar Analysen Systeme GmbH, Germany) was used for the determination of C, H, and N contents (Laboratory of Elemental Analysis at Faculty of Chemistry, University of Wrocław).

Luminescence spectra and emission lifetimes at 300 and 10 K were recorded on an FSL980 spectrofluorometer from Edinburgh Instruments. The luminescence spectra were corrected for the instrument response. A 450 W xenon arc lamp or a microsecond flashlamp (μF920) was used as an excitation source. The fluorescence quantum yield of compounds was determined at ambient temperature using an FLS980 equipped with a 150 mm integrating sphere.

#### Synthesis of [Cu(dmbpy)(μ-PTA)]_*n*_[PF_6_]_*n*_ (**1**)

Compound **1** was synthesized via LAG. A stoichiometric amount (ratio 1:1:1) of [Cu(MeCN)_4_][PF_6_] (0.32 mmol, 119 mg), PTA (0.32 mmol, 53 mg), and dmbpy (0.32 mmol, 58.9 mg) was placed in a mortar and ground for 3 min in the presence of 200 μL of MeCN. The microcrystalline yellow solid was washed with a water/methanol mixture and dried in the air. Yield based on [Cu(MeCN)_4_][PF_6_] is ca. 91%. X-ray quality yellow crystals were grown by slow evaporation of the acetonitrile solution **1** in air at room temperature. **1** is soluble in H_2_O (*S*_25 °C_ = 3 mg·mL^–1^), DMSO, MeCN, and MeOH. C_18_H_24_CuN_5_P_2_F_6_ (MW 549.90 + 4MeCN): C, 43.73; N, 17.65; H, 5.01. Found (**1**): C, 43.81; N, 17.21; H, 5.14. IR (KBr, cm^–1^): 3435(s), 2921(w), 1600(w), 1572(w), 1555(w), 1479(m), 1469(m), 1448(w), 1416(w), 1391(w), 1297(m), 1244(s), 1006(m), 1043(w), 1016(s), 970(s), 952(s), 838(vs) (PF_6_), 744(w), 731(w), 695(w), 652(w), 607(w), 583(m), 558(m), 451(w). ^1^H NMR (600.15 MHz, DMSO-*d*_6_): δ 8.64 (br s, 2H, ^6,6^H, dmbpy), 8,44 (br d, 2H, ^3,3′^H, *J*_*3–4 ’*_= 7.2 Hz, dmbpy), 7.56 (br d, 2H, ^4,4’^H, *J*_*4–3 ’*_= 7.2 Hz, dmbpy), 4.52 and 4.40 (2d, 6H, *J*_*AB*_ = 12.4 Hz, NC*H*^A^*H*^B^N, PTA); 4.12 br (s, 6H, P-CH_2_–N, PTA), 2.44 (s, 6H, ^5,5′^H, CH_3,_ dmbpy). ^31^P{H} NMR (242.95 MHz, DMSO-*d*_6_): δ −92.0 (s, PTA), −144.2 (septet, *J*_P–F_ = 710.6 Hz, PF_6_).

#### Synthesis of [Cu(bpy)(*μ-*PTA)]_*n*_[PF_6_]_*n*_ (**2**)

Compound **2** was synthesized following a procedure described for **1**, using bpy (0.32 mmol, 50 mg) instead of dmpby. Yield based on [Cu(MeCN)_4_][PF_6_] is ca. 84%. X-ray quality yellow crystals were grown by slow evaporation of the acetonitrile solution of **2** in air at room temperature. **2** is soluble in H_2_O (*S*_25 °C_ = 5 mg·mL^–1^), DMSO, MeCN, and MeOH. C_16_H_22_CuN_5_P_2_F_6_ (**2**) (MW 521.85 + H_2_O): C, 35.59; N, 12.97; H, 4.12. Found (**2**): C, 35.19; N, 13.11; H, 4.61 FT-IR (KBr, cm^–1^): 3414(m), 2929(w), 1601(m), 1593(m), 1576(w), 1497(w), 1472(m), 1446(s), 1421(m), 1365(w), 1314(w), 1298(m), 1241(s), 1178(w), 1162(w), 1109(m), 1061(w), 1038(w), 1018(vs), 988(w), 968(vs), 950(vs), 942(w), 895(w), 839(vs) (PF_6_), 790(w), 765(s), 73(w), 663(w), 650(w), 608(w), 593(w), 576(w), 558(vs), 474(w), 447(w), 415(w). ^1^H NMR (300 MHz, DMSO-*d*_6_): δ 8.76 (d, 2H, ^6,6’^H, ^3^*J*_6–5_= 4.7 Hz, bpy), 8.46 (d, 2H, ^3,3′^H, ^3^*J*_3–4_ = 8.0 Hz, bpy), 8.09 (ddd, 2H, ^4,4’^H, ^3^*J*_4–5_ = ^3^*J*_4–3_ = 8.0 Hz, ^4^*J*_4–6_ = 1.6 Hz, bpy), 7.61 (ddd, 2H, ^5,5′^H, ^3^*J*_5–6_= 4.7 Hz, ^3^*J*_5–4_= 8.0 Hz, ^4^*J*_5–3_= 1.0 Hz, bpy), 4.61 and 4.42 (2d, 6H, *J*_AB_ = 12.0 Hz, NC*H*^A^*H*^B^N, PTA), 4.32 (d, 6H, ^2^*J*_P–H_ = 2.5 Hz PCH_2_N, PTA).^31^P{^1^H} NMR (121.49 MHz, DMSO-*d*_6_): δ −73.3 (br. s), δ −144.0 (septet, ^1^*J*_F–P_ = 710.6 Hz).

#### Synthesis of [Cu(ncup)(μ-PTA)]_*n*_[PF_6_]_*n*_ (**3**)

**3** was synthesized following a procedure described for **1**, using necup (0.32 mmol, 66.64 mg) instead of dmbpy. Yield based on [Cu(MeCN)_4_][PF_6_] ca. 92%. **3** is soluble in H_2_O (*S*_25 °C_ = 1 mg·mL^–1^), DMSO, MeCN, and MeOH. C_18_H_24_CuN_5_P_2_F_6_ (MW 573.92 + CH_3_CN+H_2_O): C, 40.59; N, 11.91 H, 4.80. Found (**3**): C, 40.84; N, 12.62; H, 4.59. IR (KBr, cm^–1^): 3435(s), 3083(w), 2927(m), 2907(m), 1624(w), 1592(s), 1565(w), 1510(s), 1501(s), 1469(w), 1446(m), 1422(s), 1414(w), 1378(m), 1359(s), 1295(m), 1239(s), 1226(w), 1213(w),1155(m), 1106(s), 1037(w), 1016(vs), 983(w), 970(vs), 951(vs), 842(vs) (PF_6_), 812(w), 790(m), 774(w), 754(w), 729(m), 682(w), 653(w), 600(s), 574(m), 569(m), 558 (vs), 475(m). ^1^H NMR (600.15 MHz, DMSO-*d*_6_): δ 8.68 (d, 2H, ^7,4^H, ^3^*J*_3–4_= 8.4 Hz, ncup), 8.11 (s, 2H, ^6,5^H, ncup), 7.97 (d, 2H, ^8,3^H, ^3^*J*_3–4_= 8.4 Hz, ncup), 4.54 and 4.40 (2d, 6H, *J*_*AB*_ = 11.0 Hz, NC*H*^A^*H*^B^N, PTA), 4.16 (s, 6H, PCH_2_N, PTA), 2.96 (s, 6H, CH_3_, ncup). ^31^P{^1^H} NMR (242.95 MHz, DMSO-*d*_6_): δ −94.15 (s, PTA), 144.24 (septet, *J*_P–F_ = 710.6 Hz, PF_6_).

#### Synthesis of [Cu(phen)(*μ-*PTA)]_*n*_[PF_6_]_*n*_ (**4**)

Compound **4** was synthesized following a procedure described for **1**, but using phen (0.32 mmol, 63 mg) instead of dmbpy, yield based on [Cu(MeCN)_4_][PF_6_] ca. 95%. X-ray quality, dark-orange crystals were grown by slow evaporation of the acetonitrile solution of **4** in the air at room temperature. **4** is soluble in H_2_O (*S*_25 °C_ = 4 mg·mL^–1^), DMSO, MeCN, and MeOH. C_18_H_22_CuN_5_OP_2_F_6_ (**4**) (MW 563.88, **4**+H_2_O): C, 38.34; N, 12.41; H, 3.93. Found (**4**): C, 38.35; N, 12.34; H, 4.09. IR (KBr, cm^–1^): 3436(s), 2917(w), 1624(w), 1510(w), 1472(w), 1451(w), 1424(s), 1365(w), 1300(w), 1240(m), 1224(w), 1148(w), 1109(w), 1097(w), 1051(w), 1038(w), 1018(s), 989(w), 969(s), 952(m), 945(m), 892(w), 868(w), 837(vs), 812(w), 792(w), 770(m), 753(w), 727(s), 608(m). ^1^H NMR (600.15 MHz, DMSO-*d*_6_): δ 9.21 (br s, 2H, ^2,9^H, phen), 8.83 (br d, ^4,7^H, 2H, ^3^*J*_*3–4*_ = 6.4 Hz, phen), 8.24 (br s, 2H, ^5,6^ H, phen), 8.06 (br s, 2H, ^3,8^H, phen), 4.50 oraz 4.40 (2d, 6H, *J*_AB_ = 12.3 Hz, NC*H*^A^*H*^B^N, PTA), 4.14 (s, 6H, PCH_2_N, PTA). ^31^P{^1^H} NMR (242.95 MHz, DMSO-*d*_6_): δ −92.1 (s, PTA), −144.2 (septet, *J*_P–F_ = 710.6 Hz, PF_6_).

#### Wet Synthesis of Compounds **1**–**4**

A stoichiometric amount (ratio 1:1:1) of [Cu(MeCN)_4_][PF_6_] (0.32 mmol, 119 mg), PTA (0.32 mmol, 53 mg), and N–N-ligand (namely, dmbpy, bpy, necup, and phen, respectively, for compounds **1**–**4**) (0.32 mmol, 50–66.5 mg) was placed in a round-bottom flask and dissolved in MeCN/MeOH mixture (40 mL/15 mL). The brown solution was stirred at room temperature for 1.5 h, resulting in the formation of yellow or orange solution (color depending on the N–N-ligand used) and microcrystalline, yellow (for **1**–**3**), or orange (for **4**) solids. Yields for **1**–**4** based on [Cu(MeCN)_4_][PF_6_] are 25–40%. The obtained solution was filtered off, and the filtrate was left in a vial to slowly evaporate in the air at r.t. Orange or yellow crystals (including those of X-ray quality) were formed within 7 days. The colorless crystals of byproduct [Cu(PTA)_4_][PF_6_]·MeCN have also formed within 5 to 7 days.

### Stability Tests

Compounds in the solid state were stored in open air for over 2 years without undergoing decomposition. The PXRD studies were also performed under an inert atmosphere. Furthermore, when these compounds were dissolved in deuterated solvents in the presence of air, their NMR spectra remained unchanged after 1 week.

### X-ray Crystallography

Single-crystal data collection was performed on a XtaLAB Synergy R DW system with a HyPix-Arc 150 detector using ω-scan and Cu Kα (λ = 1.54184 Å) radiation for crystal **1** and on a Kuma diffractometer (Oxford Diffraction) with Sapphire2 CCD detector using ω-scan and Mo Kα (λ = 0.71073 Å) radiation for crystals **2** and **4**. All crystals were measured at 100 K. Cell refinement, data reduction, analysis, and absorption correction were carried out with CrysAlis PRO (Rigaku Oxford Diffraction) software [Rigaku OD (2019). CrysAlis PRO. Rigaku Oxford Diffraction Ltd., Yarnton, England]. The structure was solved by direct methods with SHELXT and refined with full-matrix least-squares techniques on *F*^2^ with SHELXL-2018/3.^61,62^ All hydrogen atoms bound to C atoms were placed in the geometrically idealized positions and treated in riding mode, with C–H = 0.95 Å and U_iso_(H) = 1.2U_eq_(C) for C–H groups and C–H = 0.98 Å and Ui_so_(H) = 1.5U_eq_(C) for CH_3_ groups.

#### Crystal Data for **1**

[CuC_18_H_24_N_5_P]PF_6_, *M* = 549.90, *a* = 20.533(6) Å, *b* = 15.592(4) Å, *c* = 6.526(3) Å, *V* = 2089.3(13) Å^3^, *T* = 100(2)K, space group *Pna*2_1_, *Z* = 4, 12689 reflections measured, 3550 independent reflections (*R*_int_ = 0.0334). The final *R*_1_ values were 0.0337 (*I >* 2σ(*I*)). The final *wR*(*F*^2^) values were 0.0883 (*I >* 2σ(*I*)). The final *R*_1_ values were 0.0351 (all data). The final *wR*(*F*^2^) values were 0.0891 (all data). CCDC 2249859 (**1**).

#### Crystal Data for **2**

[CuC_16_H_20_N_5_P]PF_6_, *M* = 521.85, *a* = 19.618(9) Å, *b* = 6.519(4) Å, *c* = 14.953(8) Å, *V* = 1912.3(18) Å^3^, *T* = 100(2) K, space group *Pnma*, *Z* = 4, 14029 reflections measured, 2741 independent reflections (*R*_int_ = 0.1131). The final *R*_1_ values were 0.1016 (*I* > 2σ(*I*)). The final *wR*(*F*^2^) values were 0.2344 (*I* > 2σ(*I*)). The final *R*_1_ value was 0.1365 (all data). The final *wR*(*F*^2^) value was 0.2470 (all data). CCDC 2249860 (**2**).

#### Crystal Data for **4**

[CuC_18_H_16_N_5_P]PF_6_, *M* = 541.84, *a* = 19.186(5) Å, *b* = 6.496(3) Å, *c* = 16.001(5) Å, *V* = 1994.2(12) Å^3^, *T* = 100(2) K, space group *Pnma*, *Z* = 4, 32348 reflections measured, 5251 independent reflections (*R*_int_ = 0.0778). The final *R*_1_ values were 0.1086 (*I* > 2σ(*I*)). The final *wR*(*F*^2^) values were 0.2839 (*I* > 2σ(*I*)). The final *R*_1_ values were 0.1256 (all data). The final *wR*(*F*^2^) value was 0.2920 (all data). CCDC 2249861 (**4**).

## Supporting Information Available

PXRD patterns and packing plots (Figures S1–S6), IR spectra (Figures S7–S10), NMR spectra (Figures S11–S18), luminescence data (Figures S19–S23), and UV monitoring of the LAG synthesis of **1**, **2**, and **4** (Figures S24–S26) (PDF)
