# Supplementary material for: Strategy for an Effective Eco-Optimized Design of Heteroleptic Cu(I) Coordination Polymers Exhibiting Thermally Activated Delayed Fluorescence
Source: Inorg Chem. 2023 Nov 27;62(49):19898–907. doi: 10.1021/acs.inorgchem.3c01908 (PMC10716906; doi:10.1021/acs.inorgchem.3c01908)
Supplement: Supplementary file 1 — ic3c01908_si_001.pdf [file ic3c01908_si_001.pdf]

Supporting Information

Strategy for an Effective Eco-Optimized Design of Heteroleptic Cu(I) Coordination Polymers Exhibiting Thermally Activated Delayed Fluorescence

Sabina W. Jaros, Jerzy Sokolnicki, Miłosz Siczek and Piotr Smoleński*

Faculty of Chemistry, University of Wrocław, F. Joliot-Curie 14, 50-383, Wrocław, Poland

E-mail: piotr.smolenski@uwr.edu.pl

Table of Contents

Figure S1. A projection along [001] of part of the crystal structure of compound **1**............3
Figure S2. Packing plot of **2** 3
Figure S3. Packing plot of **4** 3
Figure S4. PXRD patterns of compound **1**............4
Figure S5. PXRD patterns of compound **2**............4
Figure S6. PXRD patterns of compound **4**............5
Figure S7. IR spectrum of **1**............6
Figure S8. IR spectrum of **2**............6
Figure S9. IR spectrum of **3**............7
Figure S10. IR spectrum of **4**............7
Figure S11. ^{1}H NMR spectrum of **1** 8
Figure S12. ^{1}H NMR spectrum of **2**. 8
Figure S13. ^{1}H NMR spectrum of **3** 9
Figure S14. ^{1}H NMR spectrum of **4** 9
Figure S15. ^{31}P{^{1}H} NMR spectrum of **1** 10
Figure S16. ^{31}P{1H} NMR spectrum of **2**............10
Figure S17. ^{31}P{^{1}H} NMR spectrum of **3** 11
Figure S18. ^{31}P{^{1}H} NMR spectrum of **4**. 11
Figure S19. Luminescence spectra of **1** powder recorded at ambient and 10 K 12
Figure S20. Luminescence spectra of **2** powder recorded at ambient and 10 K. 12
Figure S21. Luminescence spectra of **3** powder recorded at ambient and 10 K 13
Figure S22. Luminescence spectra of **4** powder recorded at ambient and 10 K 13
Figure S23. Microsecond photoluminescence decay dynamics of **1**-**4** recorded at 300 K. 14
Figure S24. UV-monitoring of the LAG synthesis of **1**. 15
Figure S25. UV-monitoring of the LAG synthesis of **2**. 15
Figure S26. UV-monitoring of the LAG synthesis of **4**. 16

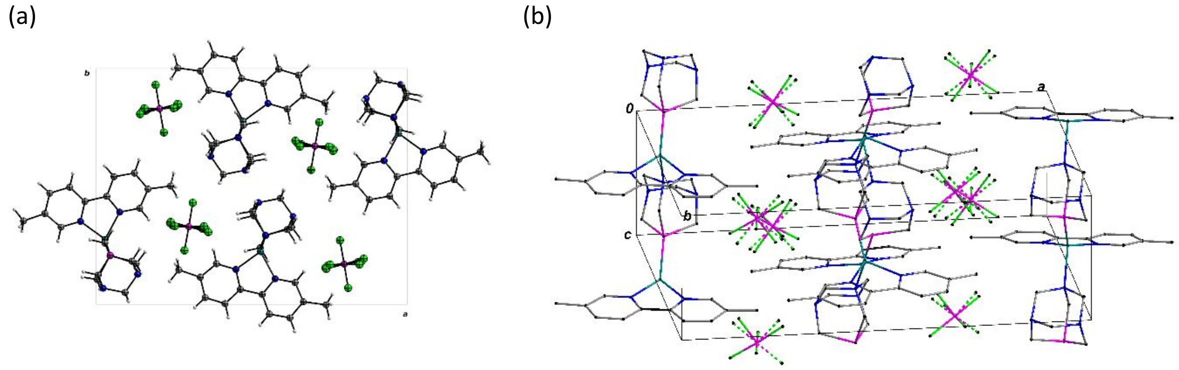

Figure S1. (a) A projection along [001] of part of the crystal structure of compound **1**. (b) Part of the crystal structure of compound **1** showing the formation of the 1D coordination polymer along [001]. The disordered component is drawn using broken lines. The hydrogen atoms have been omitted.

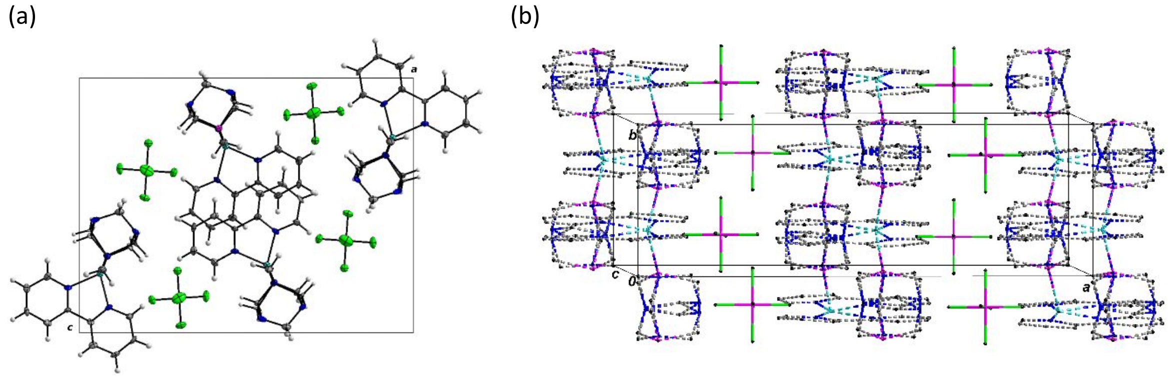

Figure S2. (a) Packing plot of **2** viewed down the b-axis direction. (b) Part of the crystal structure of compound **2** showing the formation of the 1D coordination polymer along [010]. The disordered over two position with occupation factor 0.5 PTA and bpy ligands are drawn using broken lines. The hydrogen atoms have been omitted.

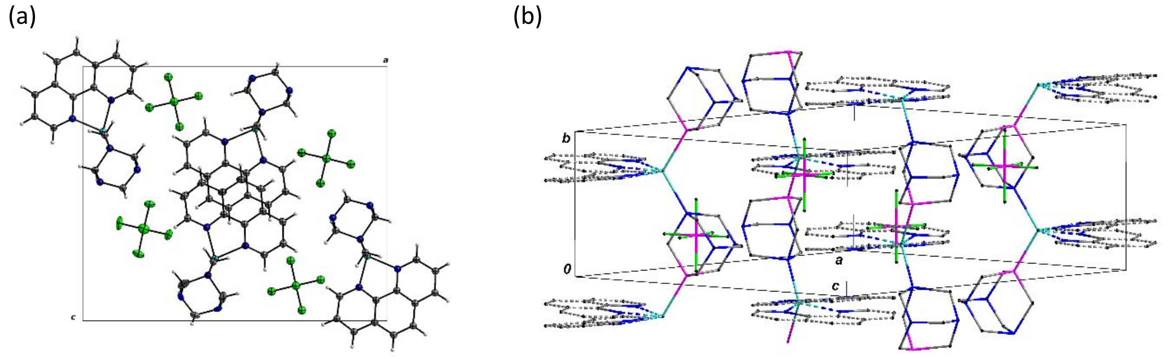

Figure S3. (a) Packing plot of **4** viewed down the b-axis direction. (b) Part of the crystal structure of compound **4** showing the formation of the 1D coordination polymer along [010]. The disordered over two position with occupation factor 0.5 phen ligand is drawn using broken lines. The hydrogen atoms have been omitted.

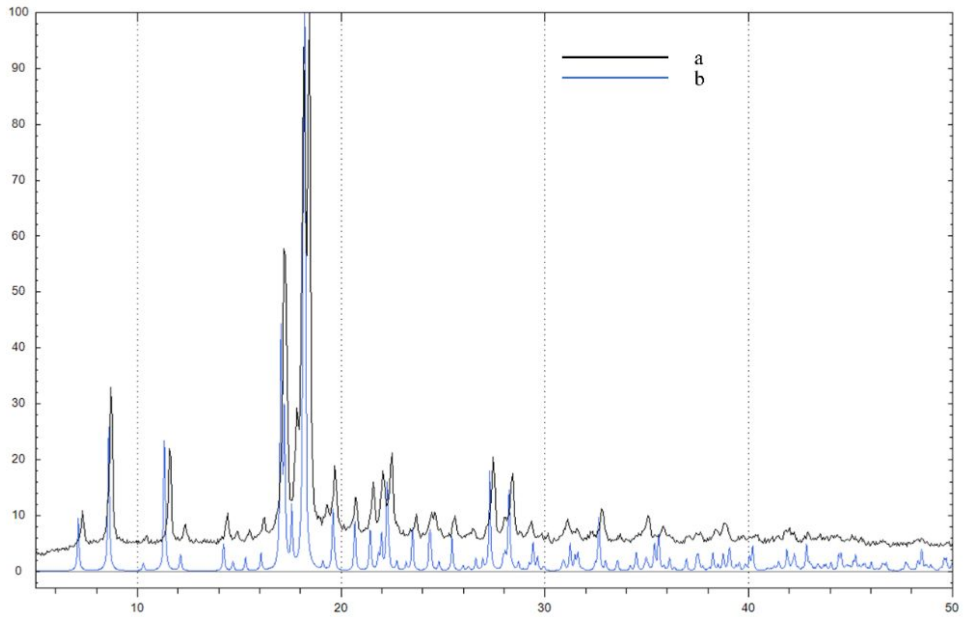

Figure S4. PXRD patterns of compound **1**: (a) bulk microcrystalline product, (b) calculated from the single crystal structure.

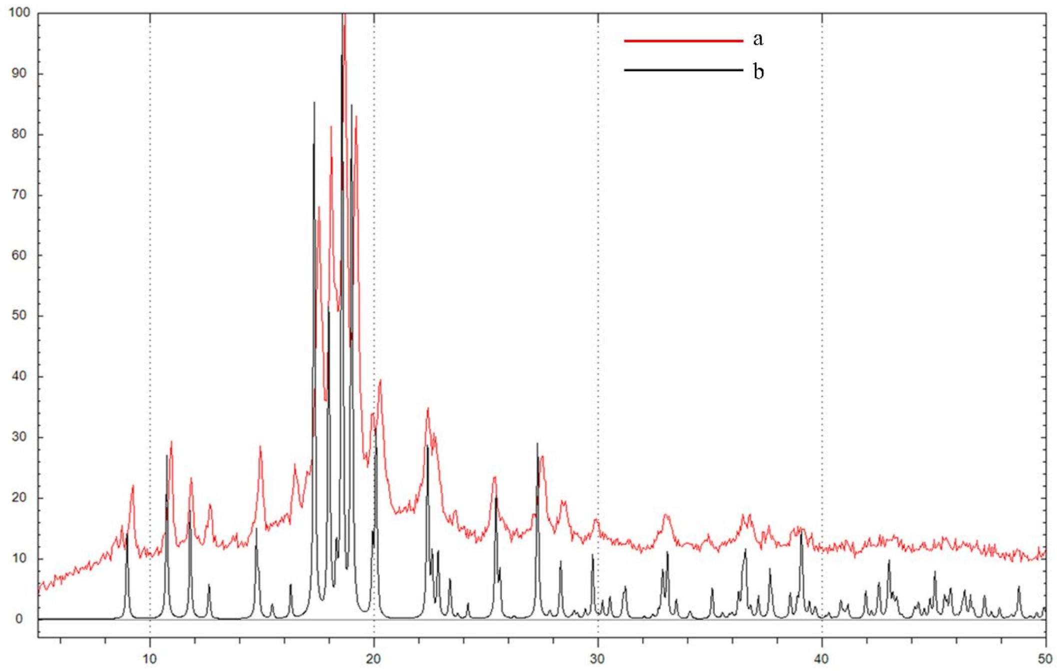

Figure S5. PXRD patterns of compound **2**: (a) bulk microcrystalline product, (b) calculated from the single crystal structure.

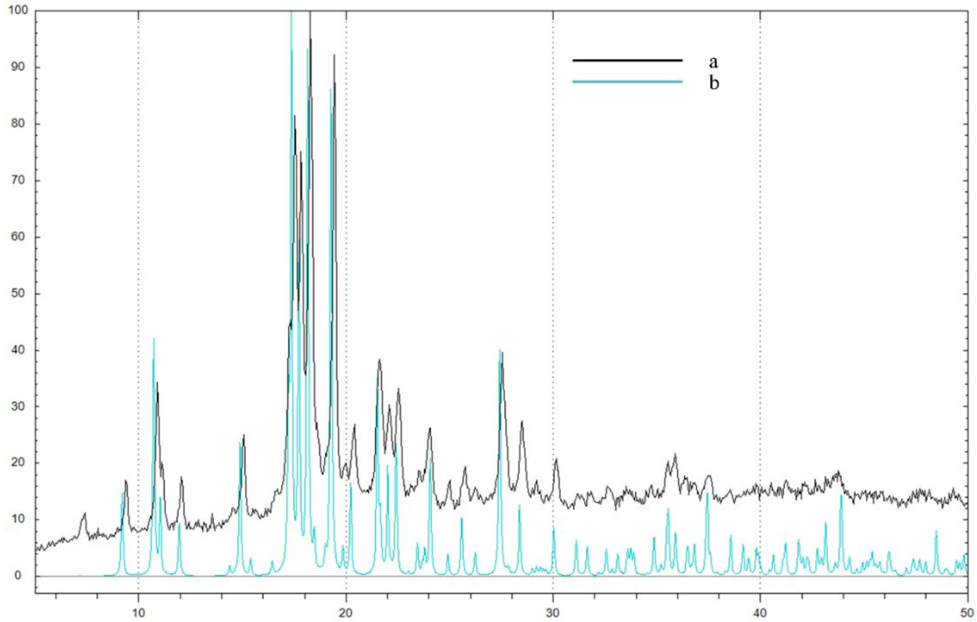

Figure S6. PXRD patterns of compound **4**: (a) bulk microcrystalline product, (b) calculated from the single crystal structure.

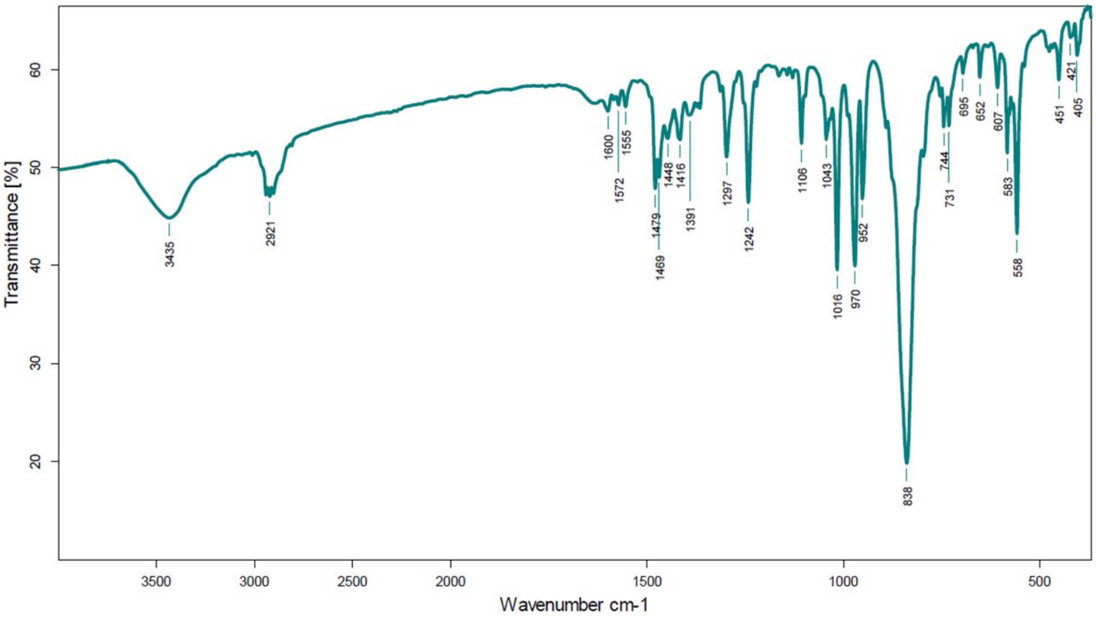

Figure S7. IR spectrum of **1**.

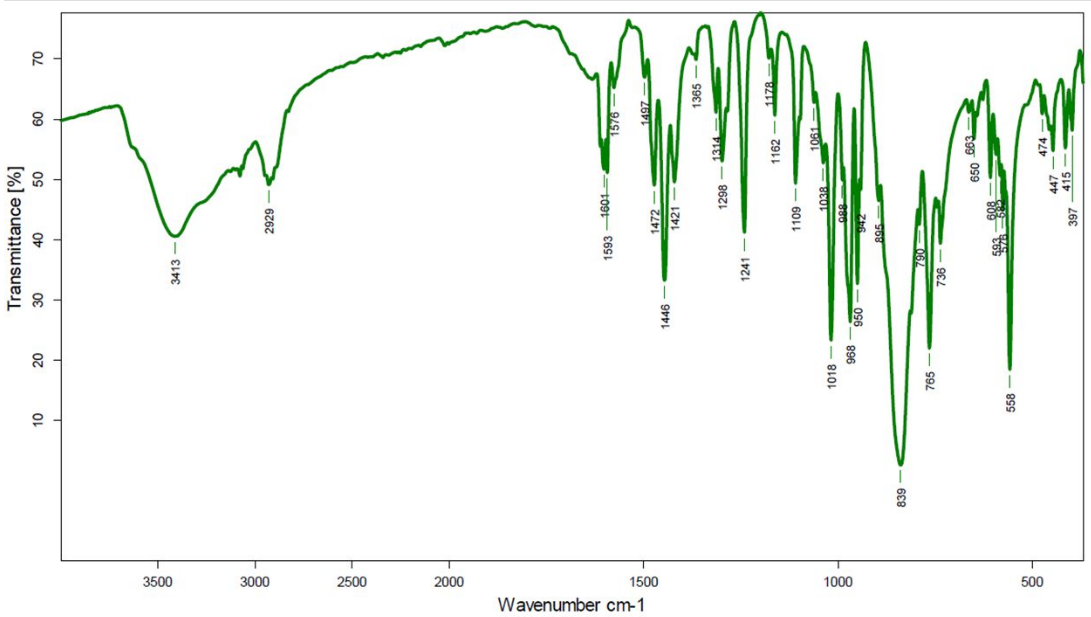

Figure S8. IR spectrum of **2**.

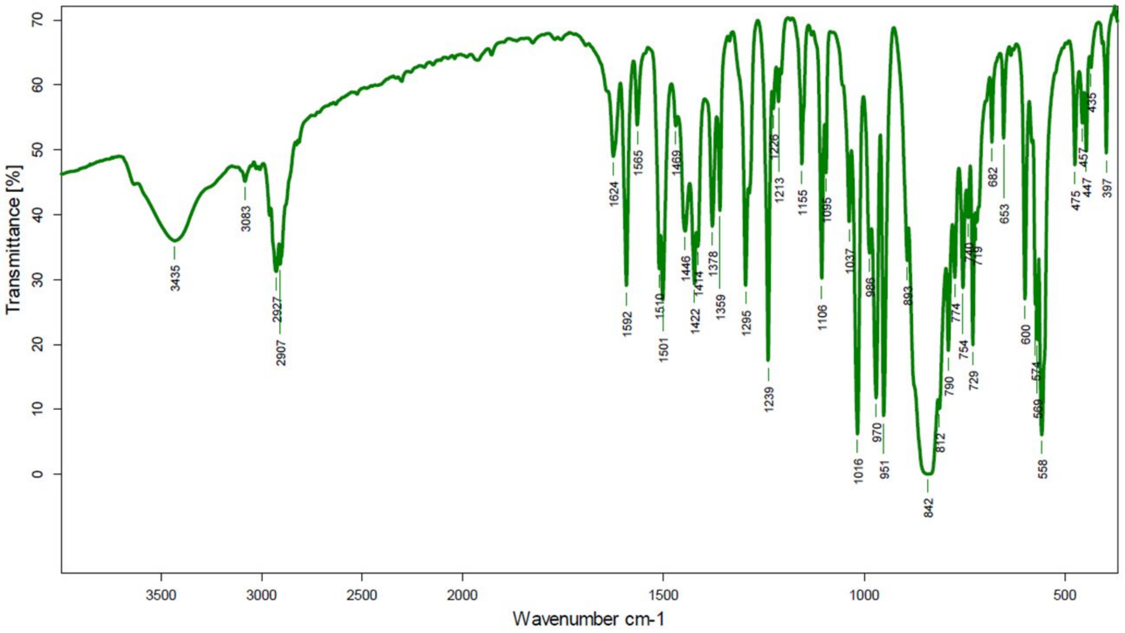

Figure S9. IR spectrum of **3**.

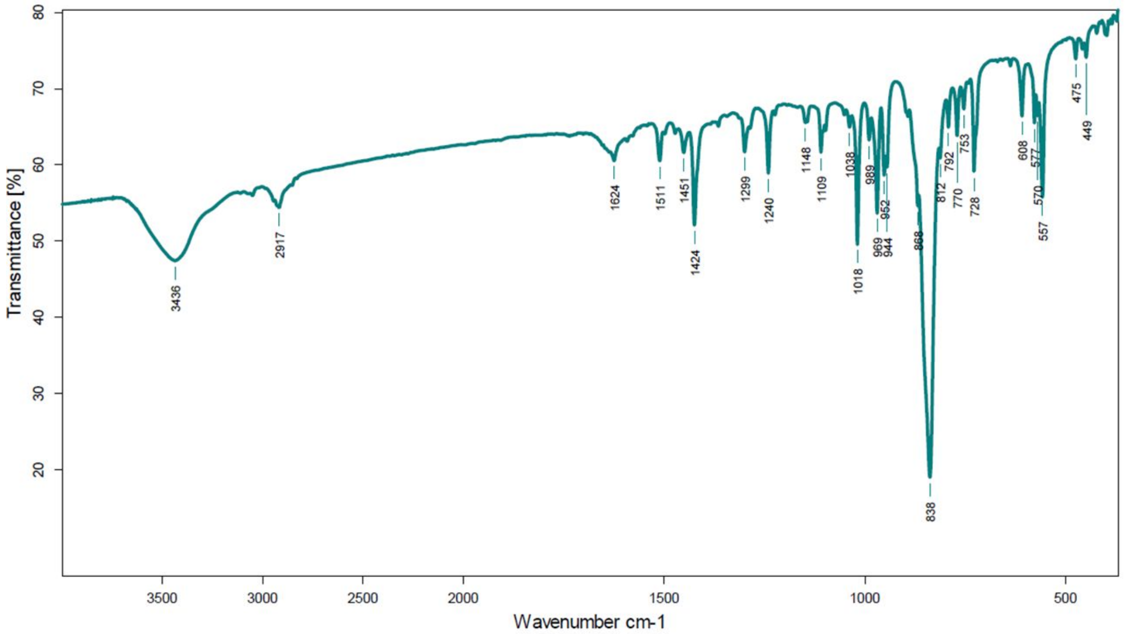

Figure S10. IR spectrum of **4**.

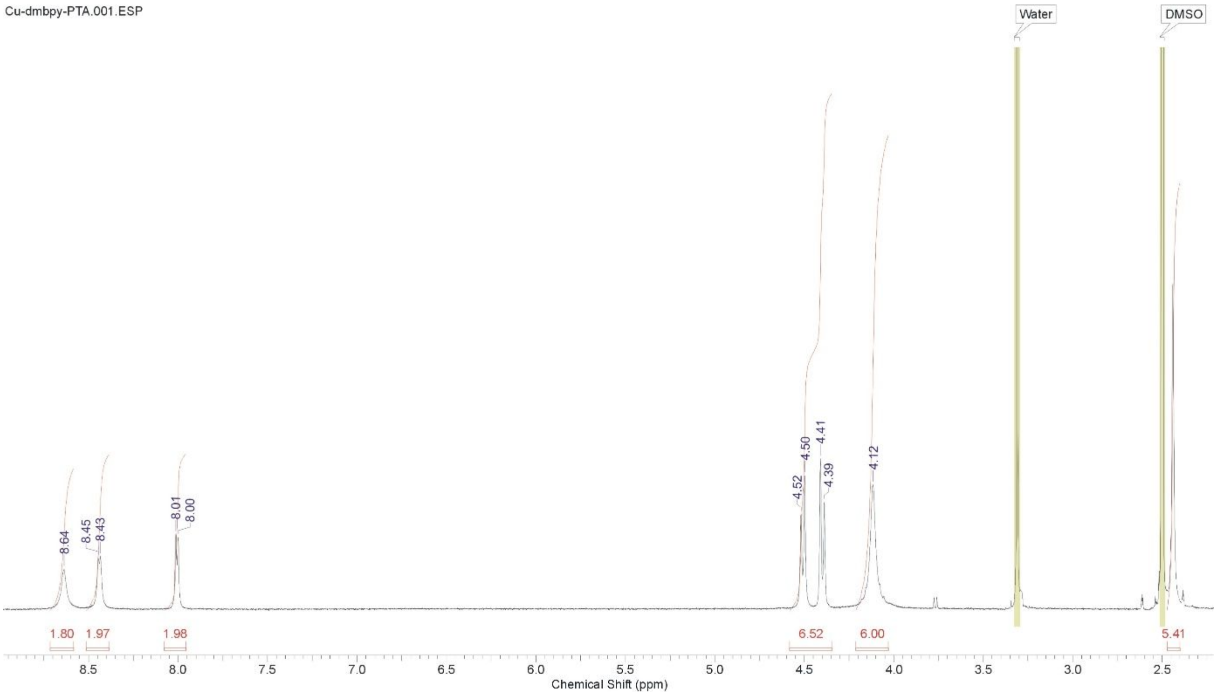

Figure S11. ^{1}H NMR spectrum of **1** in DMSO-d_6.

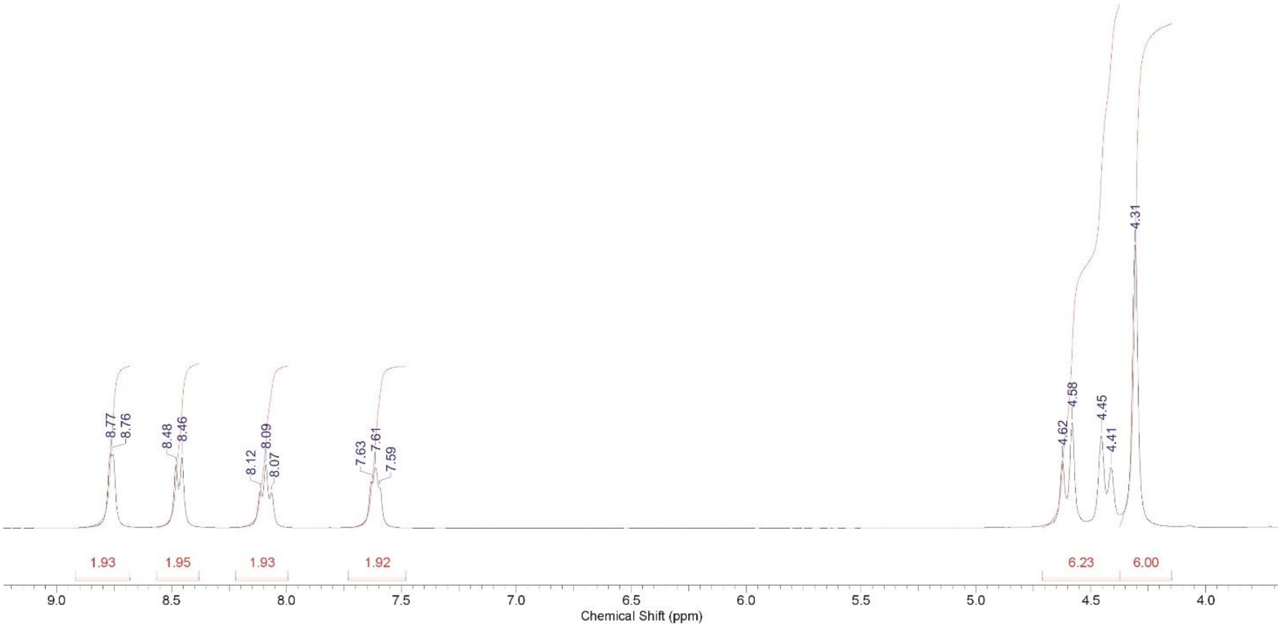

Figure S12. ^{1}H NMR spectrum of **2** in DMSO-d_6.

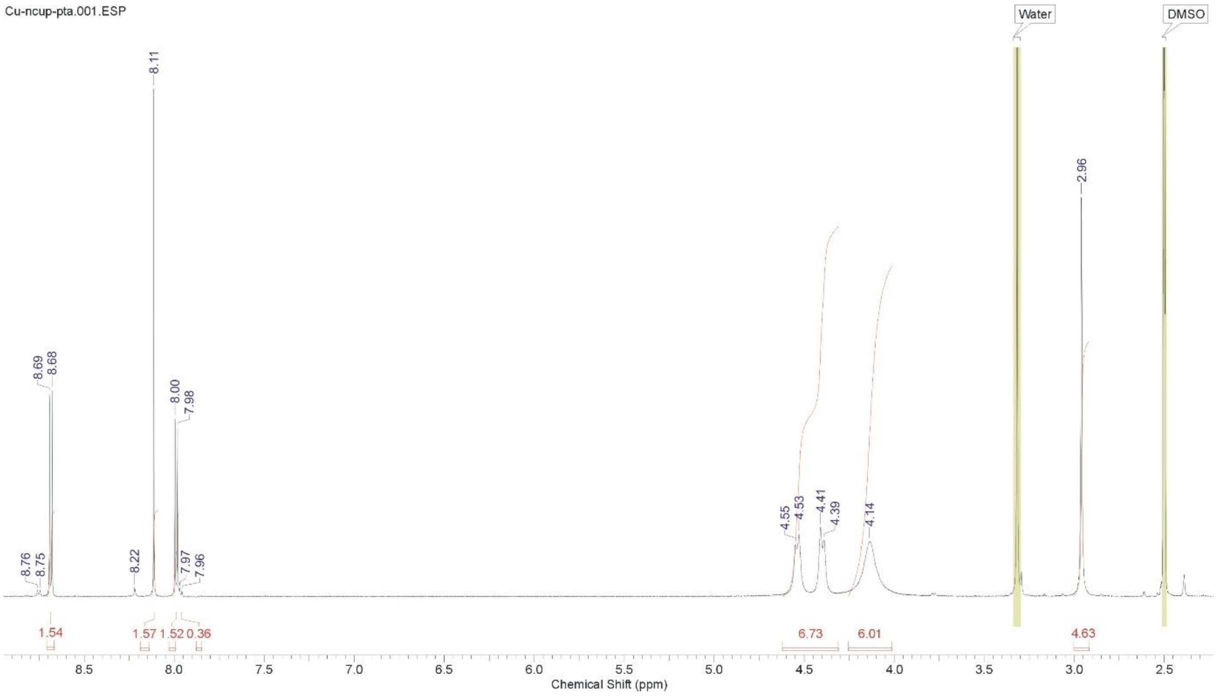

Figure S13. ^{1}H NMR spectrum of **3** in DMSO-d_6.

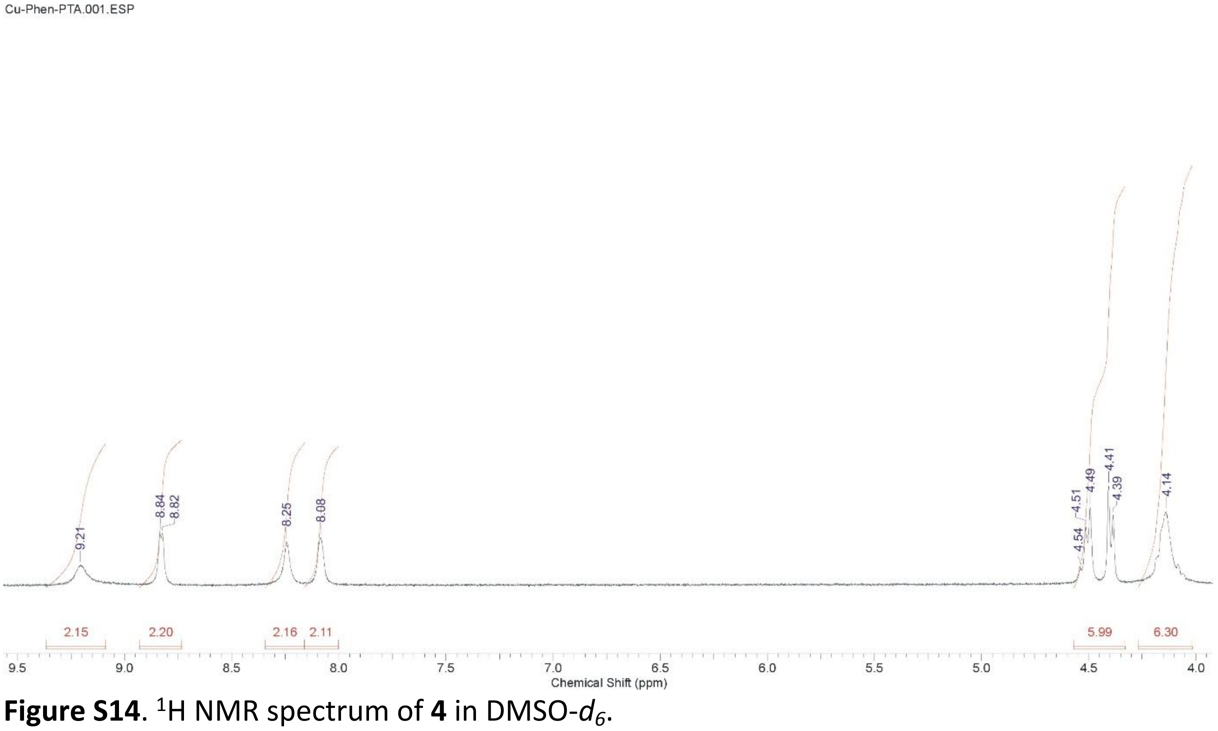

Figure S14. ^{1}H NMR spectrum of **4** in DMSO-d_6.

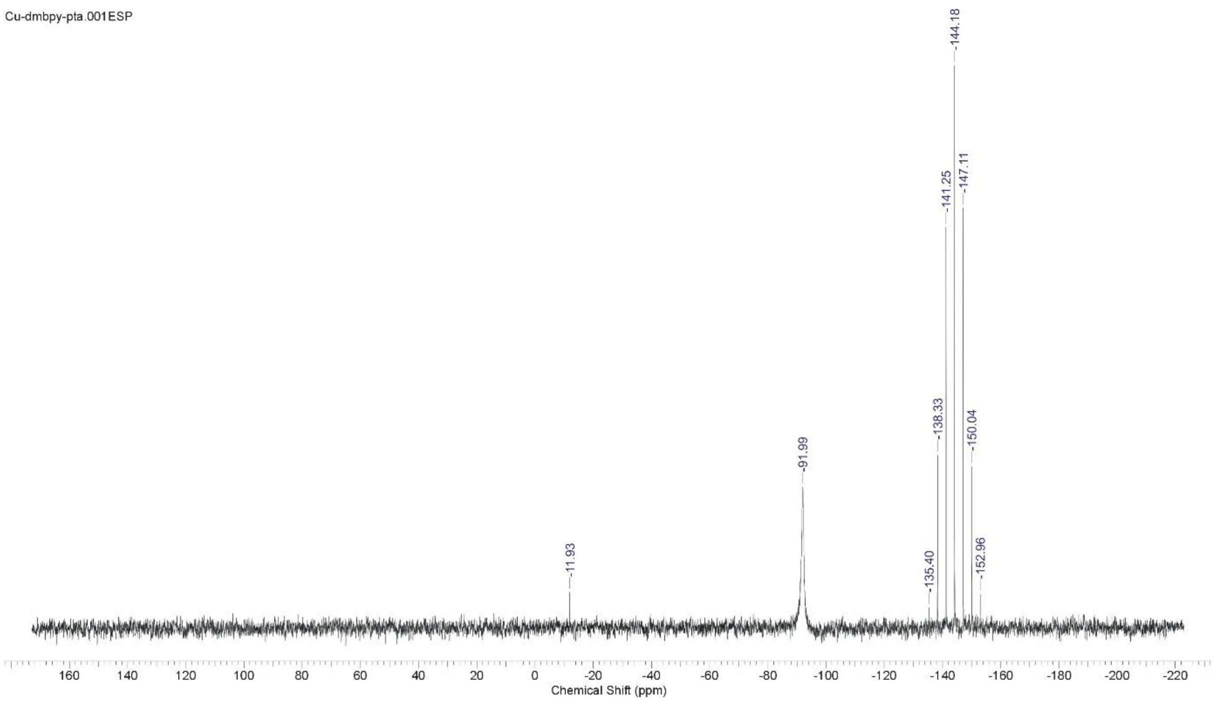

Figure S15. $^{31}P\{^1H\}$ NMR spectrum of **1** in DMSO-d_6.

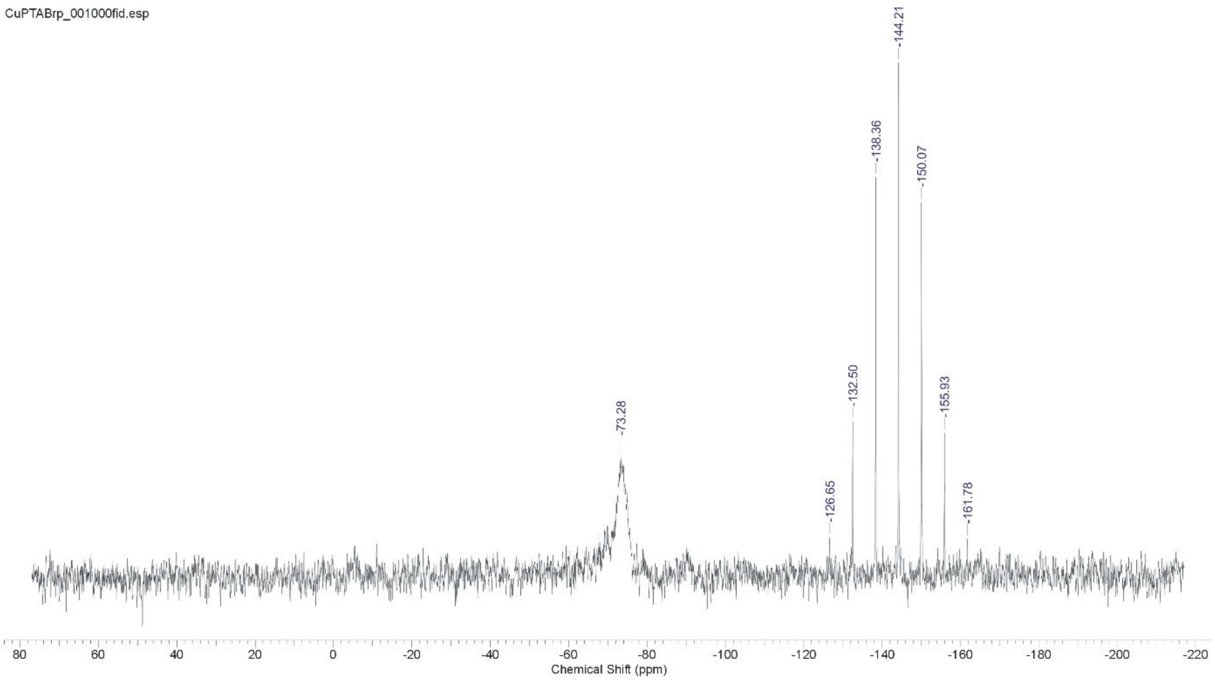

Figure S16. $^{31}P\{1H\}$ NMR spectrum of **2** in DMSO-d_6.

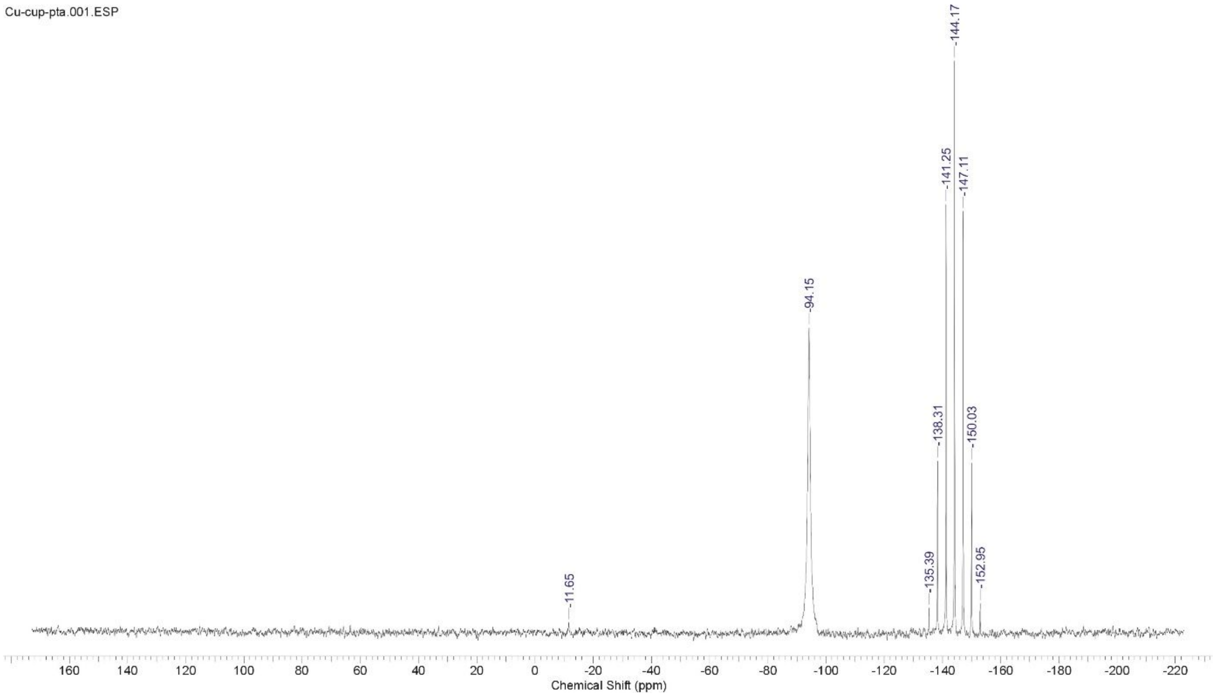

Figure S17. $^{31}P\{^{1}H\}$ NMR spectrum of **3** in DMSO-d_6.

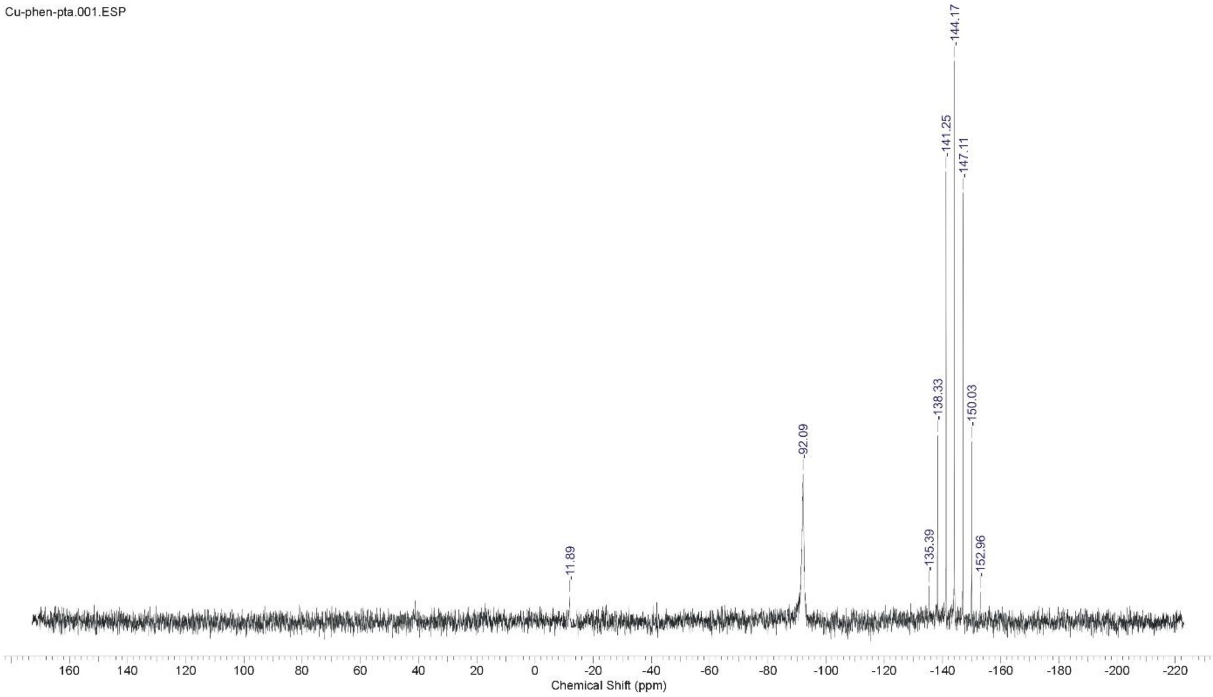

Figure S18. $^{31}P\{^{1}H\}$ NMR spectrum of **4** in DMSO-d_6.

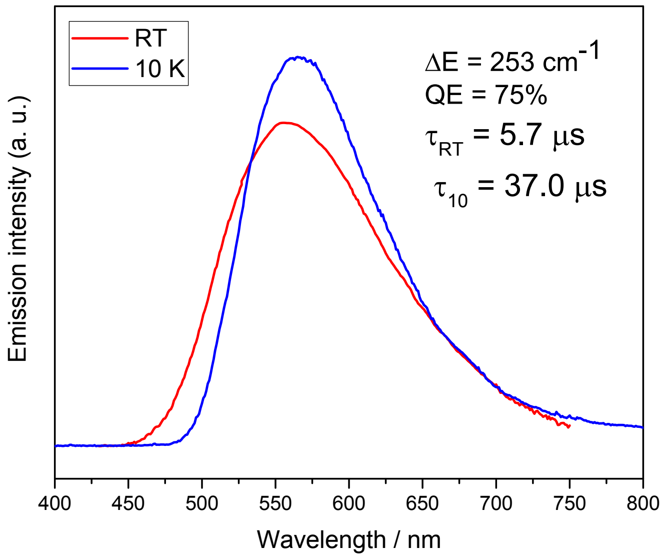

Figure S19. Luminescence spectra of **1** powder recorded at ambient (red traces) and 10 K (blue traces) temperatures. λexc = 375 nm.

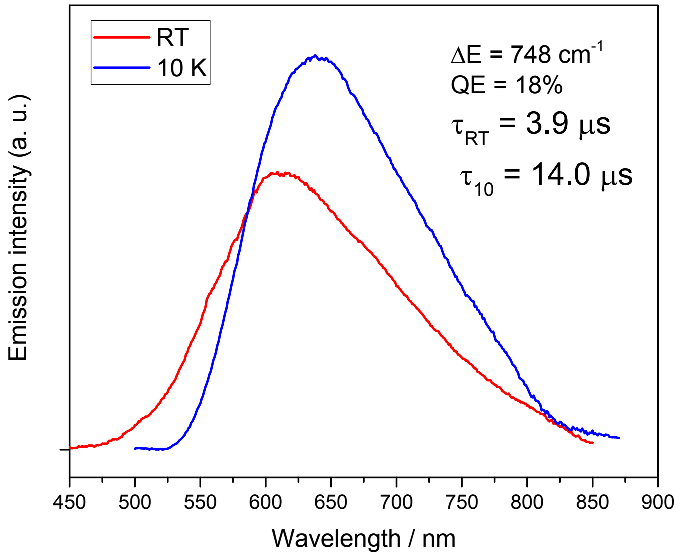

Figure S20. Luminescence spectra of **2** powder recorded at ambient (red traces) and 10 K (blue traces) temperatures. λexc = 375 nm.

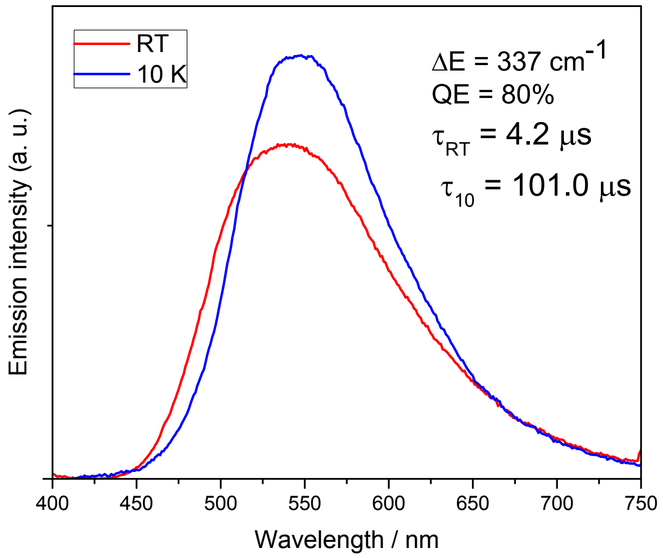

Figure S21. Luminescence spectra of **3** powder recorded at ambient (red traces) and 10 K (blue traces) temperatures. λexc = 375 nm.

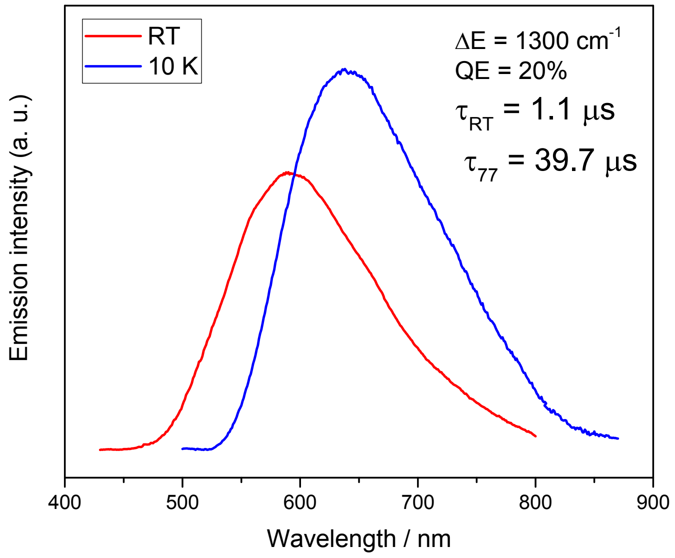

Figure S22. Luminescence spectra of **4** powder recorded at ambient (red traces) and 10 K (blue traces) temperatures. λexc = 350 nm.

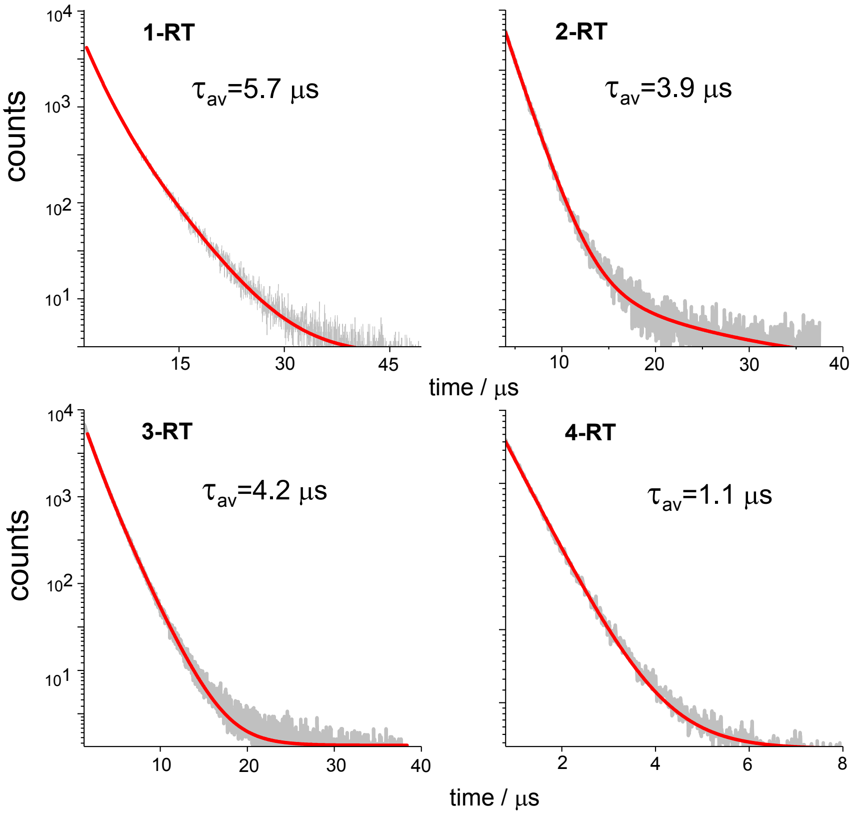

Figure S23. Microsecond photoluminescence decay dynamics of **1**-**4** recorded at 300 K. The red solid lines represent the fit to the decays.

The steady-state TADF luminescence decay dynamics at 300 K for compounds **1**-**4** show microsecond emission, fitting to a biexponential decay function.

Weighted average lifetimes values were determined by using the equation $t_{av} = \sum A_i t_i / \sum A_i$, with A_i as the pre-exponential factor for the lifetimes. The following parameters were used:

1. τ_1 = 3.3 μs, τ_2 = 6.7 μs, A_1 = 0.29, A_2 = 0.71

2. τ_1 = 1.1 μs, τ_2 = 5.5 μs, A_1 = 0.36, A_2 = 0.64

3. τ_1 = 3.1 μs, τ_2 = 6.2 μs, A_1 = 0.69, A_2 = 0.31

4. τ_1 = 0.95 μs, τ_2 = 2.5 μs, A_1 = 0.90, A_2 = 0.10

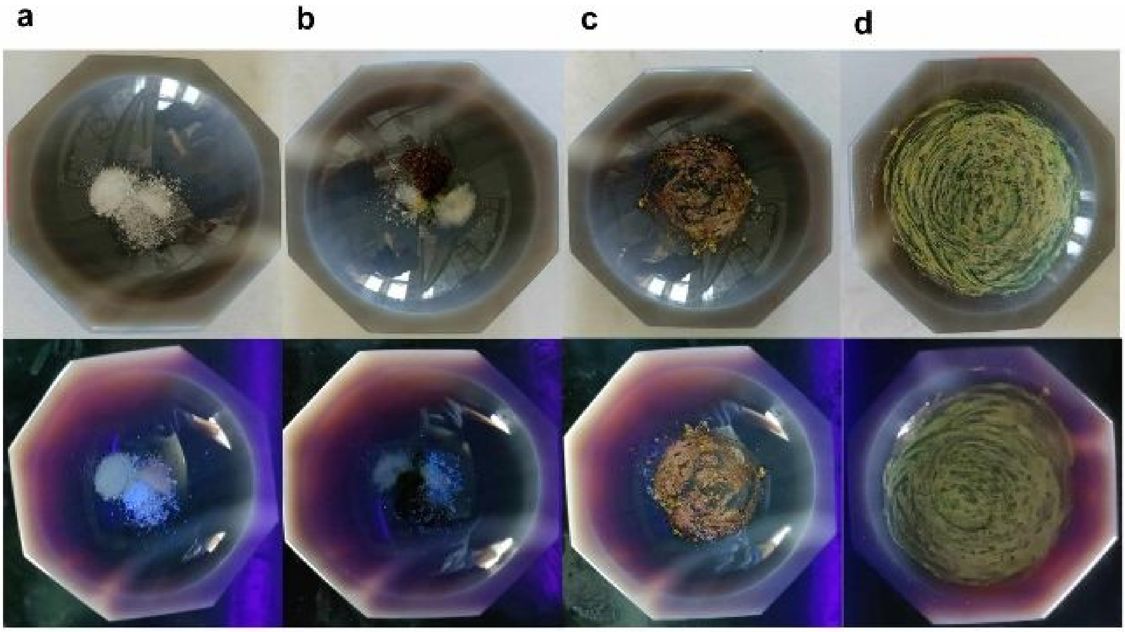

Figure S24. UV-monitoring of the LAG synthesis of **1** (down views; the top images obtained under day-light): (a) a dry mixture of $[Cu(MeCN)_4][PF_6]$ with dmbpy and PTA in 1:1:1 molar ratio; (b) the mixture with a few drops of MeCN; c) mixture after 30 s of grinding, d) ground reaction mixture after 1 minute.

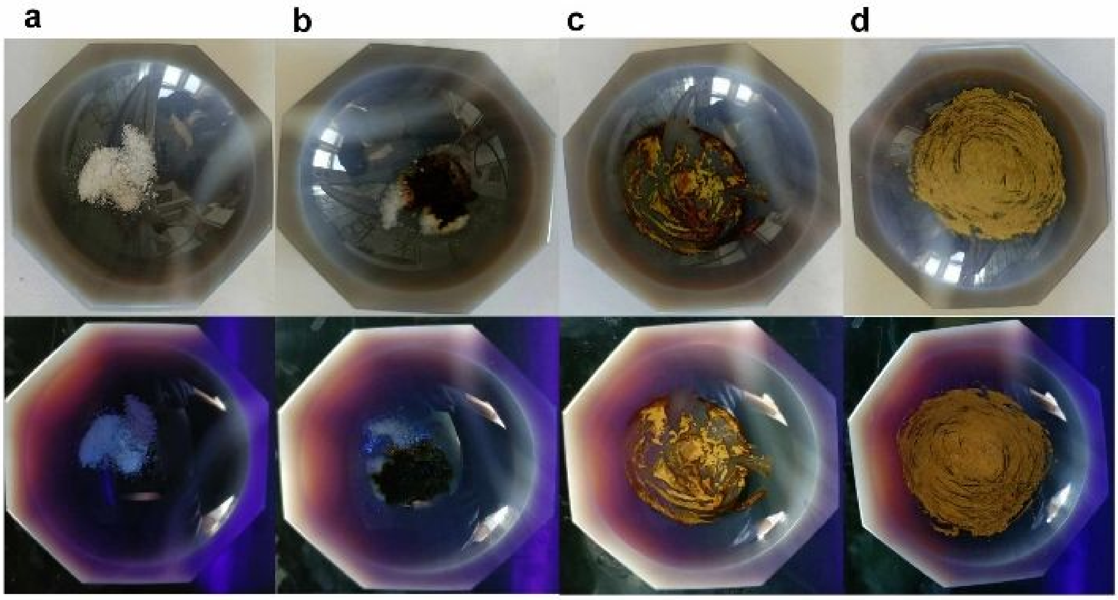

Figure S25. UV-monitoring of the LAG synthesis of **2** (down views; the top images obtained under day-light): (a) a dry mixture of $[Cu(MeCN)_4][PF_6]$ with bpy and PTA in 1:1:1 molar ratio; (b) the mixture with a few drops of MeCN; c) mixture after 30 s of grinding, d) ground reaction mixture after 1 minute.

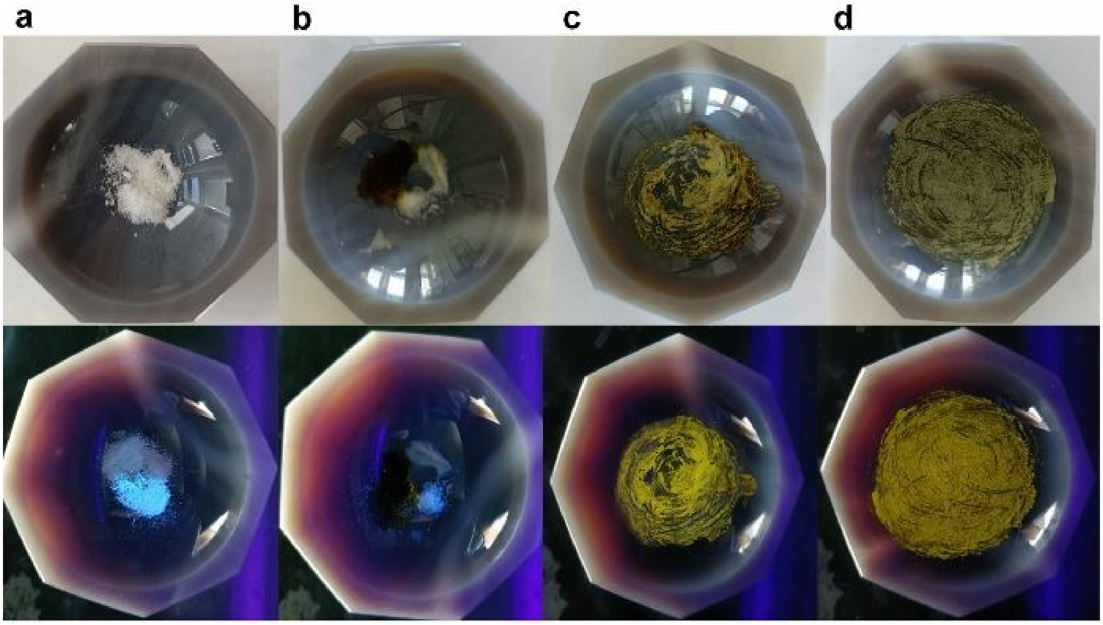

Figure S26. UV-monitoring of the LAG synthesis of **4** (down views; the top images obtained under day-light): (a) a dry mixture of $[Cu(MeCN)_4][PF_6]$ with phen and PTA in 1:1:1 molar ratio; (b) the mixture with a few drops of MeCN; c) mixture after 30 s of grinding, d) ground reaction mixture after 1 minute.
